# Depletion of tRNA CCA-adding enzyme in *Mycobacterium tuberculosis* leads to polyadenylation of transcripts and precursor tRNAs

**DOI:** 10.1038/s41598-023-47944-6

**Published:** 2023-11-24

**Authors:** Ewelina Błaszczyk, Przemysław Płociński, Ewelina Lechowicz, Anna Brzostek, Bożena Dziadek, Małgorzata Korycka-Machała, Marcin Słomka, Jarosław Dziadek

**Affiliations:** 1 Institute of Medical Biology, Polish Academy of Sciences, Lodowa 106, 93-232 Łódź, Poland; 2 Department of Immunology and Infectious Biology, Faculty of Biology and Environmental Protection, University of Łódź, Banacha 12/16, 90-237 Łódź, Poland; 3 Department of Molecular Microbiology, Faculty of Biology and Environmental Protection, University of Łódź, Banacha 12/16, 90-237 Łódź, Poland; 4 Biobank Lab, Department of Oncobiology and Epigenetics, Faculty of Biology and Environmental Protection, University of Łódź, Pomorska 139, 90-235 Łódź, Poland

**Keywords:** Microbiology, Bacterial genetics

## Abstract

In reference to gene annotation, more than half of the tRNA species synthesized by *Mycobacterium tuberculosis* require the enzymatic addition of the cytosine-cytosine-adenine (CCA) tail, which is indispensable for amino acid charging and tRNA functionality. It makes the mycobacterial CCA-adding enzyme essential for survival of the bacterium and a potential target for novel pipelines in drug discovery avenues. Here, we described the *rv3907c* gene product, originally annotated as poly(A)polymerase (*rv3907c*, PcnA) as a functional CCA-adding enzyme (CCA_*Mtb*_) essential for viability of *M. tuberculosis*. The depletion of the enzyme affected tRNAs maturation, inhibited bacilli growth, and resulted in abundant accumulation of polyadenylated RNAs. We determined the enzymatic activities displayed by the mycobacterial CCA_*Mtb*_ in vitro and studied the effects of inhibiting of its transcription in bacterial cells. We are the first to properly confirm the existence of RNA polyadenylation in mycobacteria, a previously controversial phenomenon, which we found promoted upon CCA-adding enzyme downexpression.

## Introduction

RNA processing and decay in bacteria are essential to generate functional RNAs and control the optimal level of each individual transcript. Noncoding RNAs, such as transfer RNAs (tRNAs), ribosomal RNAs (rRNAs) and small regulatory RNAs (sRNAs) usually require extensive processing during maturation to produce functional molecules. Transfer RNAs are transcribed as pre-tRNAs carrying extra nucleotides at the 5′ and 3′ termini, which are subsequently processed to produce mature tRNA by removing the extra 5′-leader and 3′-trailer regions, modifying certain nucleoside positions and synthesizing the 3′-CCA sequence, if it is not encoded by the gene, that can be aminoacylated^1–4^.

In model bacteria, the majority of tRNAs are represented in more than one copy and are often transcribed as polycistronic transcripts carrying tRNAs along with mRNAs or rRNAs and must be separated from initial premature transcripts. Usually, the processing of mono- and polycistronic transcripts is initiated by RNase E to generate pre-tRNAs containing few extra nucleotides at both the 5′- and 3′-ends. However, some polycistronic tRNA operons terminated by Rho-dependent transcription terminators in *E. coli* are processed by RNase P but not RNase E^5^. The maturation of the 5′ terminus in both gram-negative and gram-positive bacteria is catalyzed by RNase P, a very conserved endonucleolytic ribozyme^6,7^. The 3′ end processing of tRNAs, if required after RNase E and/or RNase P cleavage, involves various 3′ → 5′ exoribonucleases^4,8^. In *E. coli,* the 3′ extensions might be removed by either of the redundant enzymes: RNases T, PH, D and R; however RNases T and PH are considered the most important^2,9^. In *E. coli,* all pre-tRNAs have the CCA motif encoded within their genes and these sequences get exposed at the 3′ termini during the maturation process. However, two classes of tRNA precursors exist in gram-positive bacteria since only a portion of genes (2/3 in *B. subtilis*) carry a CCA motif encoded by tRNA genes. The key ribonuclease involved in the maturation of 3′ CCA-free termini is RNase Z which is present in many gram-positive bacteria, including *B. subtilis, Staphylococcus aureus, Streptococcus pyogenes* and *M. tuberculosis*^3^. In most cases, RNase Z cleaves tRNA precursors just downstream of the discriminatory base yielding a tRNA ready for synthesis of the CCA motif^10,11^.

The 3′ CCA sequence, which is the site for the acylation of the appropriate amino acid^12,13^, is required for proper positioning of the amino acid in the ribosomal A-site^14,15^, and assists in peptide bond formation as well as in the positioning of water molecules for nucleophilic attack in the translation termination process^16–18^. If the CCA sequence is not included in a tRNA gene, it is synthesized by essential ATP(CTP):tRNA nucleotidyltransferases-commonly known as CCA-adding enzymes (for review see^19^). In *E. coli,* where all tRNA genes encode the 3′-CCA sequence, the CCA-adding enzyme repairs and maintains the CCA termini^20–22^. Nonfunctional tRNAs have been reported to be marked by degradation tags and short poly(A) tails synthesized by poly(A) polymerase and are processed by degrading exonucleases^23–25^. Quality control might also be based on the synthesis of a secondary CCA sequence by CCA adding enzyme and the CCACCA tail is recognized as a degradation tag^26–28^.

The highly orchestrated, multistage tRNA maturation process is scarcely characterized in the major human bacterial pathogen *M. tuberculosis*. Here, we found that CCA-adding enzyme (CCA_*Mtb*_) is an essential enzyme of *M. tuberculosis* and *Mycobacterium smegmatis.* CCA_*Mtb*_ depletion was associated with a significant increase in the transcriptome polyadenylation rates, which are negligible in the wild-type strain. Amongst the most abundant polyadenylated species were the precursor tRNA molecules. We believe that CCA_*Mtb*_-mediated processes could potentially be good targets for future anti-tuberculosis drug discovery pipelines.

## Results

### Rv3907c is the CCA-adding enzyme in *Mycobacterium*

**Figure 1:**
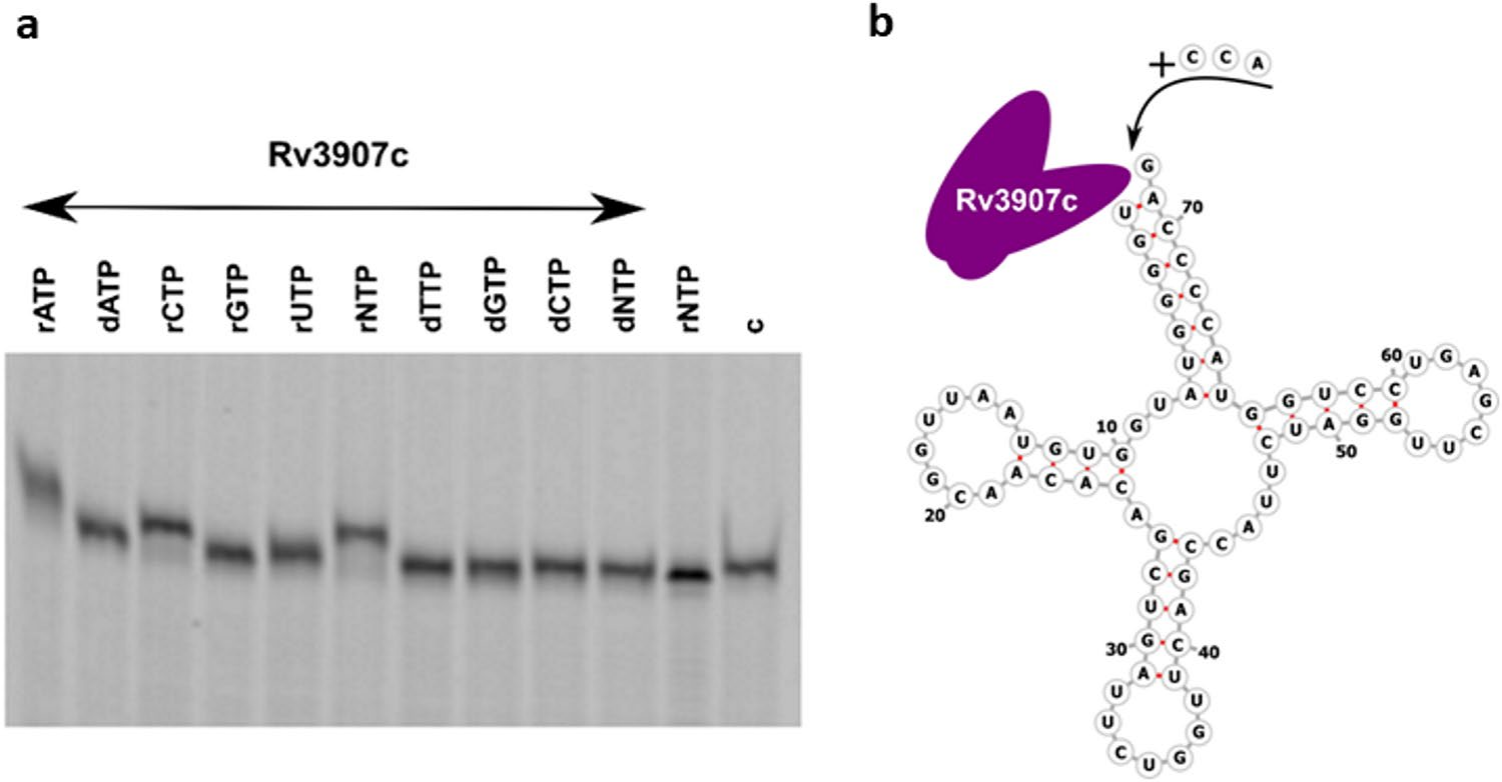
tRNAs nucleotidyltransferase activity of Rv3907c. (**a**) Gel analysis (10% acrylamide gel containing 8 M UREA) of Hex-labeled tRNAGln72 incubated in the presence of Rv3907c and various ribonucleotides or deoxyribonucleotides. Lines 1–10, rATP, dATP, rCTP, rGTP, rUTP, rNTP, dTTP, dGTP, dCTP, dNTP, respectively, Line 11—no Rv3907c control, 12, c—no nucleotides control. (**b**) Schematic representation of CCA_*Mtb*_ activity on tRNAGln72 (modified from RNAfold WebServer).

It remains unclear whether the *rv3907c* gene product, originally annotated as poly(A) polymerase, is in fact the CCA-adding enzyme in *Mtb*. Rv3907c is composed of three domains, an N-terminal class II polymerase β superfamily domain, a central RNA-binding domain and a C-terminal HD domain, specifically its HDIG variant, which is also present in ribonuclease Y. A similar HD domain is also present in the CCA-adding enzyme of *E. coli*’s but not in its poly(A) polymerase, which might indicate that the presence of a functional HD domain is important to the tRNA processing activities of enzymes from this family. Following the analysis described by^29^ we found that the poly(A) polymerases-specific upstream motif located between motif A (the common signature of all nucleotidyltransferases) and the position of the flexible loop, is absent in Rv3907c (Fig. S1). On the other hand, Rv3907c contains a flexible loop element which is not present in CC-adding enzymes, as well as, a second motif identified more downstream, ERxxxExxxhh, which distinguishes CCA-adding from A-adding (sRxxxExxxhh) enzymes^30^. While 45 different tRNA species are encoded on the genome of *Mtb*, including three variants of tRNA-Met, all 64 known codons are present and utilized in the coding sequences of the genome. According to the mycobrowser annotation (https://mycobrowser.epfl.ch/, accessed on 28th of March, 2023), out of 45 different tRNA species, the vast majority require the addition of a final or partial sequence of the terminal CCA, with only 13 tRNA species having the CCA sequence intrinsically present on their primary transcripts (Table S1). Moreover, the mycobacterial transfer-messenger RNA (tmRNA) is also the substrate for CCA adding as its genomic sequence ends with CCG and not with CCA. The presence of a tRNA nucleotidyltransferase domain, the similarity to the characterized *B. subtilis* CCA-adding enzyme, suggested the involvement of Rv3907c in the synthesis and/or repair of CCA-terminal tRNA sequences. To determine the activity of Rv3907c, the protein was expressed in *E. coli*, purified by metal-ion affinity followed by anion exchange chromatography (Fig. S2), and used in in vitro assay. Additionally, the recombinant gene variant mut-*rv3907c* was constructed and expressed in the *E. coli.* Mut-Rv3907c protein carrying substitutions of key amino acids of the tRNA nucleotidyltransferase domain (DLD/57-59/ALA) was expressed and purified alike the intact Rv3907c. Having purified the Rv3907c recombinant enzyme, we tested its activity as CCA-adding enzyme in vitro. We incubated the Hex-labeled synthetic sequence of *Mtb*’s tRNA_Gln_*,* which does not possess the natural CCA (Fig. 1b), with Rv3907c and observed the limited elongation of tRNA in the presence of Rv3907c and rATP or rCTP ribonucleotides as well as in the presence of rNTP mixture but not when rGTP or either of the deoxyribonucleotides were included in the reaction mix exclusively (Fig. 1a).

**Figure 2:**
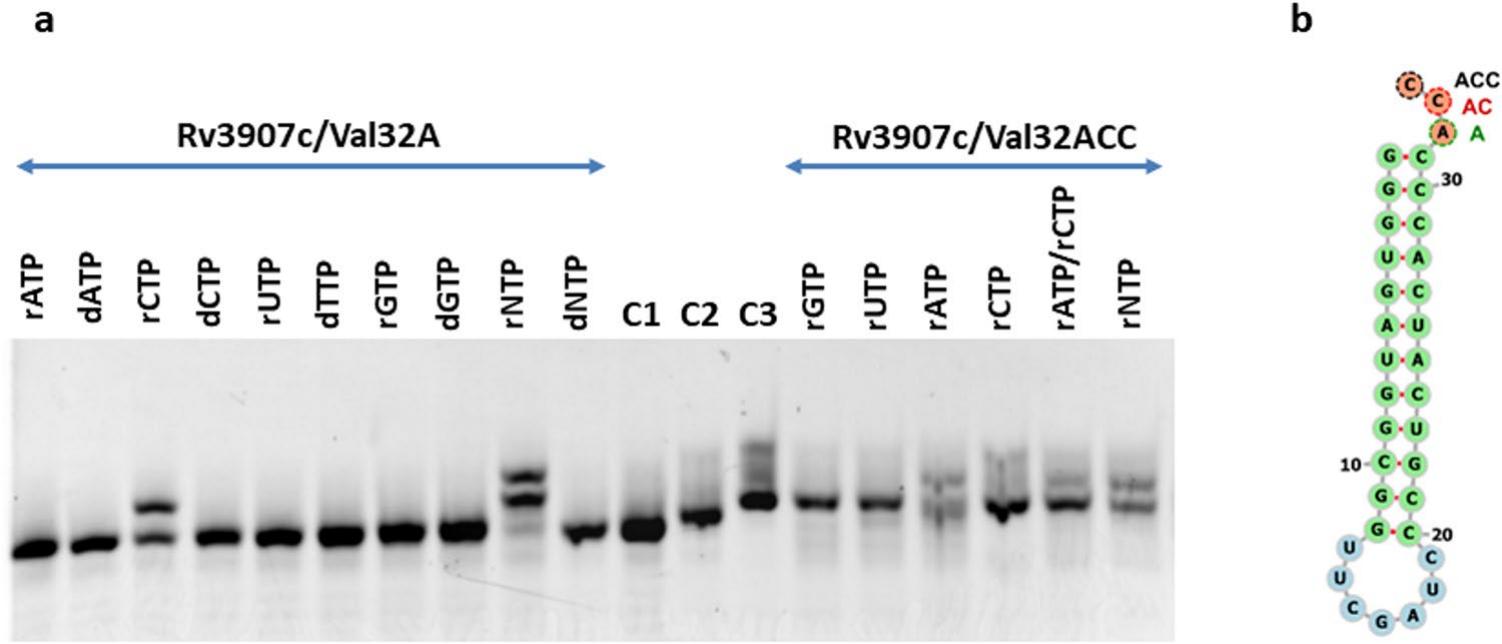
CCA-adding enzyme activity of Rv3907c. (**a**) Gel analysis (10% acrylamide gel containing 8 M UREA) of Hex-labeled tRNAs (Val32A, Val33AC, Val34ACC) incubated in the presence of CCA_*Mtb*_ and various ribonucleotides (r) and deoxyribonucleotides (d). C1 (Val32A), C2 (Val32AC), C3 (Val32ACC)—no CCA_*Mtb*_ control. The analyzes were performed at least in triplicate using independently prepared and purified CCA_*Mtb*_ enzyme. (**b**) Schematic representation of template Val32ACC (modified from RNAfold WebServer).

To further confirm the putative tRNA nucleotidyltransferase activity of Rv3907c at single nucleotide resolution, we applied a partial *E. coli* tRNA_Val_ sequence (34, 32 nucleotides in length of the 3′ end) terminated by ACC or A based on previously published observations (Fig. 2b)^31^. In the presence of Rv3907c, the A-terminated template was elongated by 2–3 nucleotides in the presence of rCTP, rNTP or rATP/rCTP but not in the presence of rATP, rGTP, rUTP or any deoxyribonucleotide. The ACC-terminated template was elongated with a single nucleotide in the presence of rATP, rATP/rCTP or rNTP, but with lower efficiency (Fig. 2a). The above-described experiments have confirmed that Rv3907c is indeed the mycobacterial tRNA nucleotidyltransferase and should be named CCA-adding enzyme (CCA_*Mtb*_).

**Figure 3:**
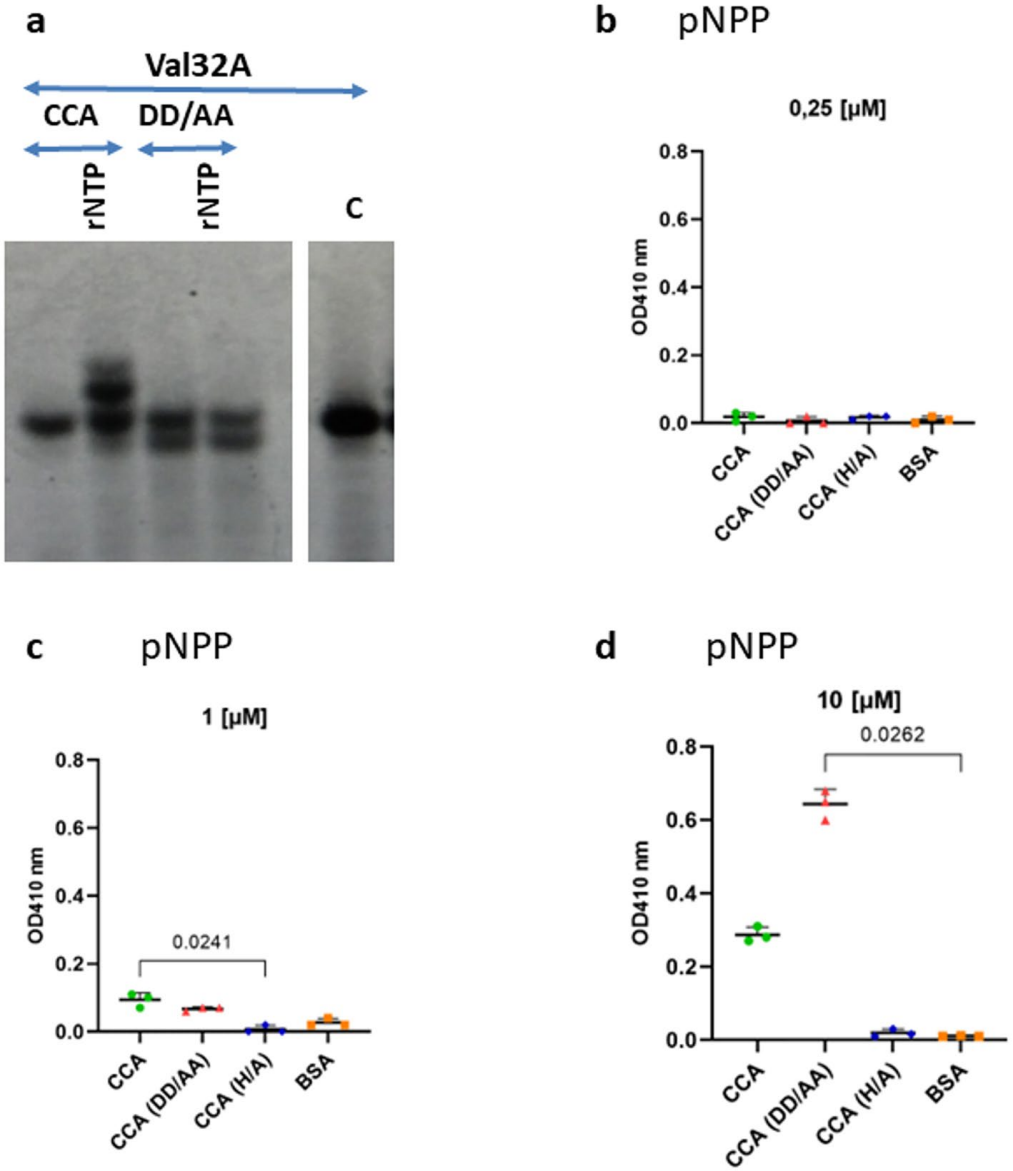
DLD/57–59/ALA affects the tRNA nucleotidyltransferase but not phosphohydrolase activity of CCA-adding enzyme. (**a**) CCA-adding enzyme activity of wild-type and DLD/57–59/ALA mutated Rv3907c. Gel analysis (10% acrylamide gel containing 8 M UREA) of Hex-labeled tRNAs (Val32A) incubated in the presence of CCA_*Mtb*_ or its mutant DLD/57–59/ALA and ribonucleotides mixture (rNTP) if indicated. C represents no protein control (see supplementary file for original blot). (**b**–**d**) The phosphohydrolase activity of CCA_*Mtb*_ and its mutated variants DD/AA (DLD/57–59/ALA) and H/A (H/298/A) in concentrations (**b**) 0.25 µM, (**c**) 1 µM, (**d**) 10 µM determined calorimetrically at 410 nm in the presence of p-nithrophenyl phosphate (pNPP). Statistical analysis was performed by Kruskal–Wallis one-way ANOVA with post-hoc Dunn’s test. The analyzes were performed in triplicate using independently prepared and purified CCA_*Mtb*_ enzymes.

Then, to determine whether the conserved amino acids, identified in the putative tRNA nucleotidyltransferase domain, are essential for the observed activity, we compared the activity of the wild-type Rv3907c enzyme and its mutant containing the DLD/57-59/ALA substitutions. Elongation of the 32-nucleotide substrate was observed exclusively in the presence of the wild-type enzyme, (Fig. 3a). On the other hand, both wild-type and DLD/57-59/ALA mutated enzymes showed the metal-dependent phosphohydrolase activity using a pNPP (p-nitrophenyl phosphate, disodium salt) hydrolysis assay. The phosphohydrolase activity of CCA_*Mtb,*_ resulting from the presence of HDIG domain, was determined calorimetrically as an increase in the optical density (OD_410_ nm) due to the enzyme-dependent hydrolysis of pNPP (Fig. 3b–d). The reactions were carried out in the presence of 0.25, 1, and 10 µM wild-type CCA_*Mtb*_, tRNA nucleotidyltransferase mutated enzyme (DLD/57-59/ALA), HDIG mutated enzyme (substitution of the key amino acid in HD domain, H/298/A), and control BSA protein (Fig. 3b–d). Compared to the control reactions supplemented with BSA, a significant increase in the optical density was observed in the reactions carrying 1 and 10 µM wild-type and DLD/57-59/ALA CCA_*Mtb*_ but not HDIG (H/298/A) mutated CCA_*Mtb*_.

### Rv3907c is essential for the viability of mycobacteria

Previous high-density transposon mutagenesis studies have classified *rv3907c* gene of *M. tuberculosis (Mtb)* as essential for in vitro growth^32,33^. We precisely verified this observation by using homologous recombination for the human pathogen *Mtb* as well as saprophytic fast-growing *M. smegmatis* (*Msm*)^34,35^ using two-step recombination protocol^36–38^. The analysis of at least 50 individual *Msm* and *Mtb* double crossover recombinants (DCO) confirmed that the knockout mutation in *rv3907c* is lethal for mycobacteria. Furthermore, to confirm the essentiality of *rv3907c,* the intact copy of the gene, under the control of the *P*_*ami*_ or *P*_*fas2*_ promoter, was introduced by site-specific recombination into the *attB* site^39,40^ of the wild type *Mtb* and *Msm* strains to support the expression of Rv3907c. The extra copy of *rv3907c* allowed us to select DCO mutants deprived of a native copy of the gene in both species (Fig. S3). Additionally, the *attB* integrated intact copy of *rv3907c* provided with the pMV306Hyg^R^ vector in both *Msm* and *Mtb* was subjected to replacement with an “empty” pMV306Kan^R^ vector in three independent experiments. The lack of Kan^R^ recombinants without intact *rv3907c* finally confirmed the essentiality of *rv3907c* for the viability of both tested mycobacterial species.

### Depletion of Rv3907c affects the growth of mycobacteria under various conditions

To determine the physiological consequences of Rv3907c depletion in slow- and fast-growing mycobacteria, we applied the CRISPRi-dCas9 system, adapted to mycobacteria^41^. The Cas9-*rv3907c-*antisense sequence, or Cas9 sequence alone as a control, was expressed from inducible (anhydrotetracycline, aTc) promoter in *Mtb* and *Msm* mutants. The aTc-dependent depletion of Rv3907c/ MSMEG_6926 (Rv3907c ortholog in *Msm*) was monitored by Western blot analysis with anti-Rv3907c polyvalent rabbit serum. The protein was depleted by at least 90% in *Mtb/Msm* Cas9-*rv3907c/ msmeg_6926-*mutants growing in media supplemented with aTc but not in the same media without inducer or in strains carrying the Cas9 sequence exclusively growing with or without aTc (Fig. S4). The depletion of Rv3907c affected the growth of mycobacteria in rich (7H9/OADC) medium at various pH values (5.7; 6.6; 7.2) as well as on minimal media supplemented with glycerol (Fig. S5). We also noted that depletion of Rv3907c affects the survival of *Mtb* and *Msm* under conditions of limited oxygen (Fig. S6).

Considering the above with Rv3907c as a reliable target for antimycobacterial compounds, we asked questions about the global response of tubercle bacilli to the depletion of Rv3907c. To address this question, we completed global RNA sequencing for *Mtb rv3907c*^*CRISPRi/dCas9*^ and *Mtb* carrying empty *CRISPRi/dCas9* vector as a control with and without aTc in three biological repeats. We initially performed total RNA sequencing using Illumina-compatible libraries. While we created two strains with two alternative *rv3907c* silencing sites, one based on a medium-strength PAM resulting in 4.39-fold downregulation of *rv3907c* mRNA (Log2FC = − 4.39; FDR > 0.05) and the second based on a weaker PAM resulting in 3.7-fold downregulation of *rv3907c* mRNA (Log2FC = − 3.70; FDR > 0.05), we noticed more transcriptional differences associated with the latter construct, which might have resulted from improved/prolonged cell viability. The depletion of Rv3907c led to alteration of the expression of 437 genes (Log2FC =|2|; FDR > 0.05). Of these 437 genes, 159 genes were downregulated, and 278 were overexpressed (Dataset S1).

### Rv3907c-depletion affects tRNA maturation in vivo and leads to transcript polyadenylation

**Figure 4:**
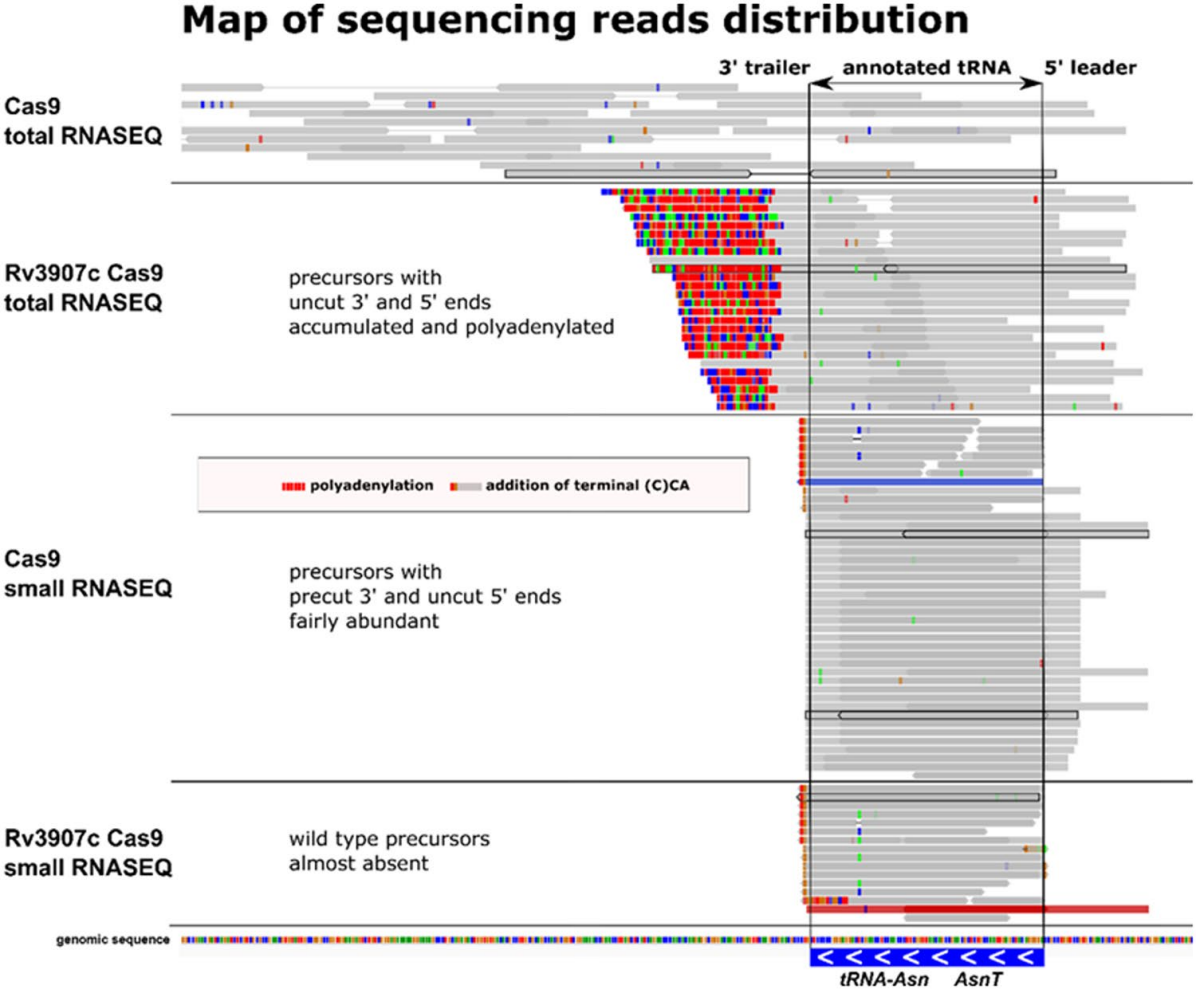
Polyadenylation of tRNA precursor molecules in vivo is associated with Rv3907c downregulation**.** Map of the transcriptomic read type and distribution aligned to genomic sequence in the vicinity of the region encoding tRNA-Asn(GTT) as an example of accumulation of polyadenylated unprocessed tRNA species. Representative sections of sequencing visualized with Integrative Genomic Viewer are presented in horizontal panels for total RNASEQ and small RNASEQ for a control strain carrying an empty Cas9 plasmid and Rv3907c Cas9 depletion strain. Typical exemplary reads in each panel are bolded.

From our total RNA-Seq analysis, we identified 18 tRNA species (out of 45) within the strongly overexpressed genes, and upon closer inspection of sequencing reads, we recognized these as accumulated unprocessed premature transcripts rather than mature tRNA molecules. Typical total RNA-Seq protocols do not allow adequate sequencing of small RNA species, which are normally depleted in such libraries, including the loss of mature tRNAs, which are only 70–80 nt in length^42^. The apparent accumulation of lengthy, premature tRNA precursors allowed their inclusion in total RNA-Seq libraries. Importantly, both 5′ and 3′ tRNA precursor ends remained unprocessed, which can likely be explained by the strictly sequential order of tRNA maturation in bacteria. Furthermore, the immature species of tRNA that accumulated upon Rv3907c depletion were strongly polyadenylated (an example shown in Fig. 4). The poly(A) tails were of varying length and rather heterogeneous, with frequent random insertions of nucleotides other than adenine.

Upon noticing extensive polyadenylation of transcripts associated with Rv3907c depletion, we wanted to confirm this observation with an alternative technique. We exploited the *Msm* strain deprived of MSMEG_6926 expression via the *CRISPRi/dCas9* approach, analogous to the *Mtb* strain described above. For constructing sequencing libraries, we used oligo-dT-coated beads to specifically preselect polyadenylated sequences (see Dataset S2). The preselection allowed us to spot some polyadenylated sequences in the empty Cas9 strain, however, these were present at very low abundance. Confirming our initial observations from *Mtb*, using oligo-dT-coated beads for polyadenylated RNA enrichment and library construction, we noted a large number of polyadenylated unprocessed tRNA precursors in the MSMEG_6926 depleted strain (Fig. S7).

**Figure 5:**
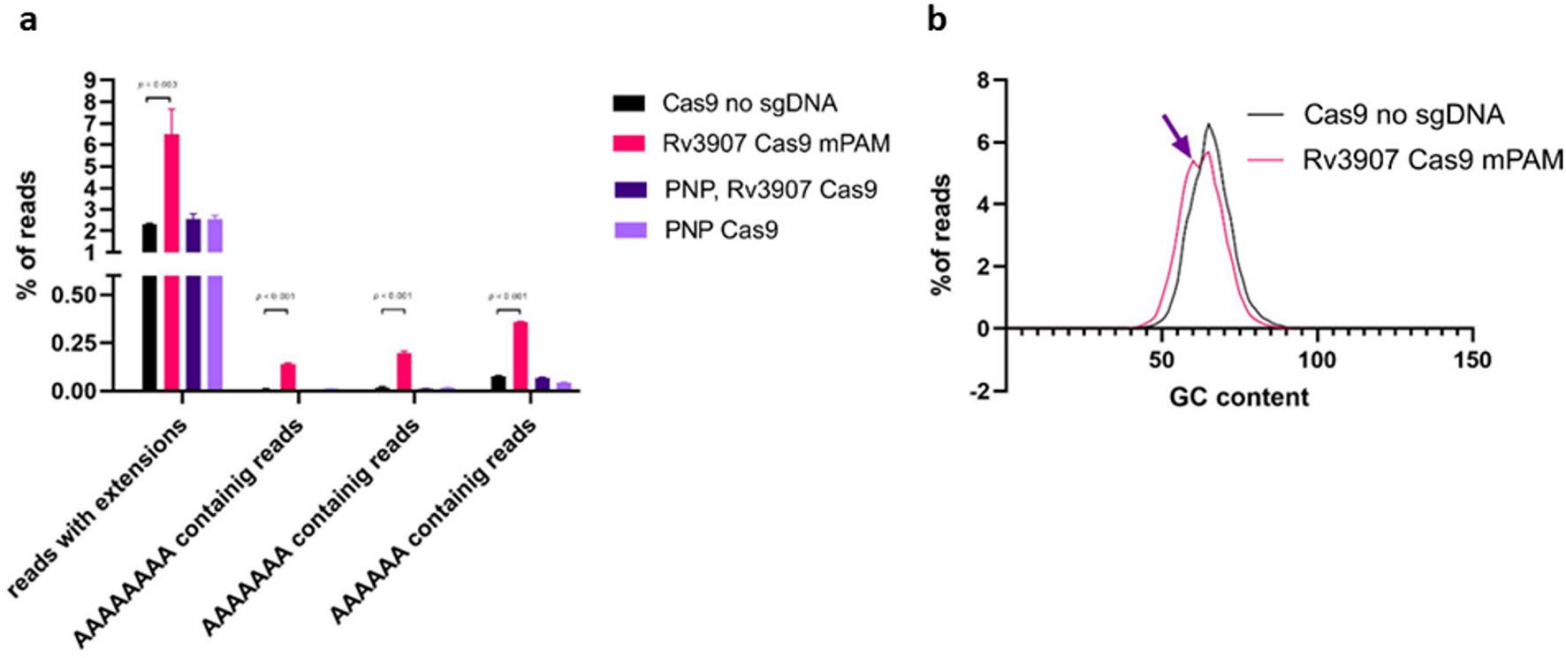
Polyadenylation rates of Cas9 regulated strains depleted of Rv3907c. (**a**) Polyadenylation rates of strains carrying Cas9 constructs targeting Rv3907c (Rv3907 Cas9 mPAM, based on^41^) were compared to Cas9 strain containing an empty pJLR966 plasmid (Cas9 no sgDNA), Rv3907c/PNP double Cas9 depletion strain (PNP, Rv3907 Cas9) and the Cas9 PNPase depletion strain (PNP Cas9, re-analyzed^43^ added to complete analysis. (**b**) GC content calculated with FastQC tool (http://www.bioinformatics.babraham.ac.uk/projects/fastqc/) for mapped sequencing reads is shown, with arrow indication of a secondary pick advocating for the presence of polyadenylated sequences.

To quantify the polyadenylated transcripts, we compared the total number of polyadenylated transcripts in wild-type and Rv3907c-depleted *Mtb* strains from total RNA sequencing data obtained without oligo-dT-coated beads enrichment. Since the poly(A) tails observed in our strains contained large number of nonadenosine nucleotides, we first extracted and quantified mapped reads which contained unaligned extensions. As *Mtb* does not encode for stretches of adenosine nucleotides on its genome, we also quantified reads containing such sequences across all sequencing reads (Fig. 5a) and calculated total GC content in Rv3907c-depleted and control *Mtb* (Fig. 5b).

The transcriptome of the wild-type strain contained around 2.2 ± 0.06% of reads carrying extensions (sequencing adapters excluded) with 0.075 ± 0.005% of total reads containing AAAAAA sequences. In contrast, the *Mtb rv3907c*^*CRISPRi/dCas9*^ mutant strain contained 6.48 ± 1.18% extended reads with 0.36 ± 0.005% of reads containing AAAAAA sequences (Fig. 5a). Inspection of GC content performed exclusively on mapped sequencing reads revealed the presence of a secondary peak of reads with lower GC content in the *rv3907c*^*CRISPRi/dCas9*^ strain when compared to the Cas9 control strain, advocating for their polyadenylation (Fig. 5b).

Knowing PNPase as the enzyme capable of polymerization of poly(A) tails, we constructed a double CRISPRi-dCas9 mutant affecting the transcription of both *rv3907c* (Rv3907c) and *gpsI* (PNPase) genes at the same time. In such mutant, depleted of both proteins (Rv3907c and PNPase) nor in a single PNPase mutant reported by us previously (reanalysed^43^) we did not observe the polyadenylation of transcripts in the total RNASeq (Fig. 5a), indicating that polyribonucleotide nucleotidyltransferase PNPase is indeed the enzyme responsible for polyadenylation of transcripts, at least in the case of Rv3907c deficiency.

To comprehensively estimate the abundance of all kinds of RNA species upon depletion of the Rv3907c enzyme, the same RNA samples that were used for total RNA sequencing, were also used to produce small RNA libraries with a dedicated kit from Illumina (Dataset S3). The estimation of differential gene expression was narrowed to annotated non-coding RNAs with the exclusion of rRNA species. The analysis of such libraries revealed 20 changes when comparing the Rv3907c depleted strain to the control strain carrying an empty Cas9 vector (Log2FC =|1,583|; FDR > 0.05). Amongst these changes, 6 were related to tRNA with apparent depletion of mature tRNA-Met(MetV, CAT) and accumulation of tRNA-Ser(SerX, CGA), tRNA-Tyr(TyrT, GTA), tRNA-Arg(ArgV, CCG), tRNA-Ser(SerU, TGA), and tRNA-Val(ValT, CAC) (Dataset S3).

**Figure 6:**
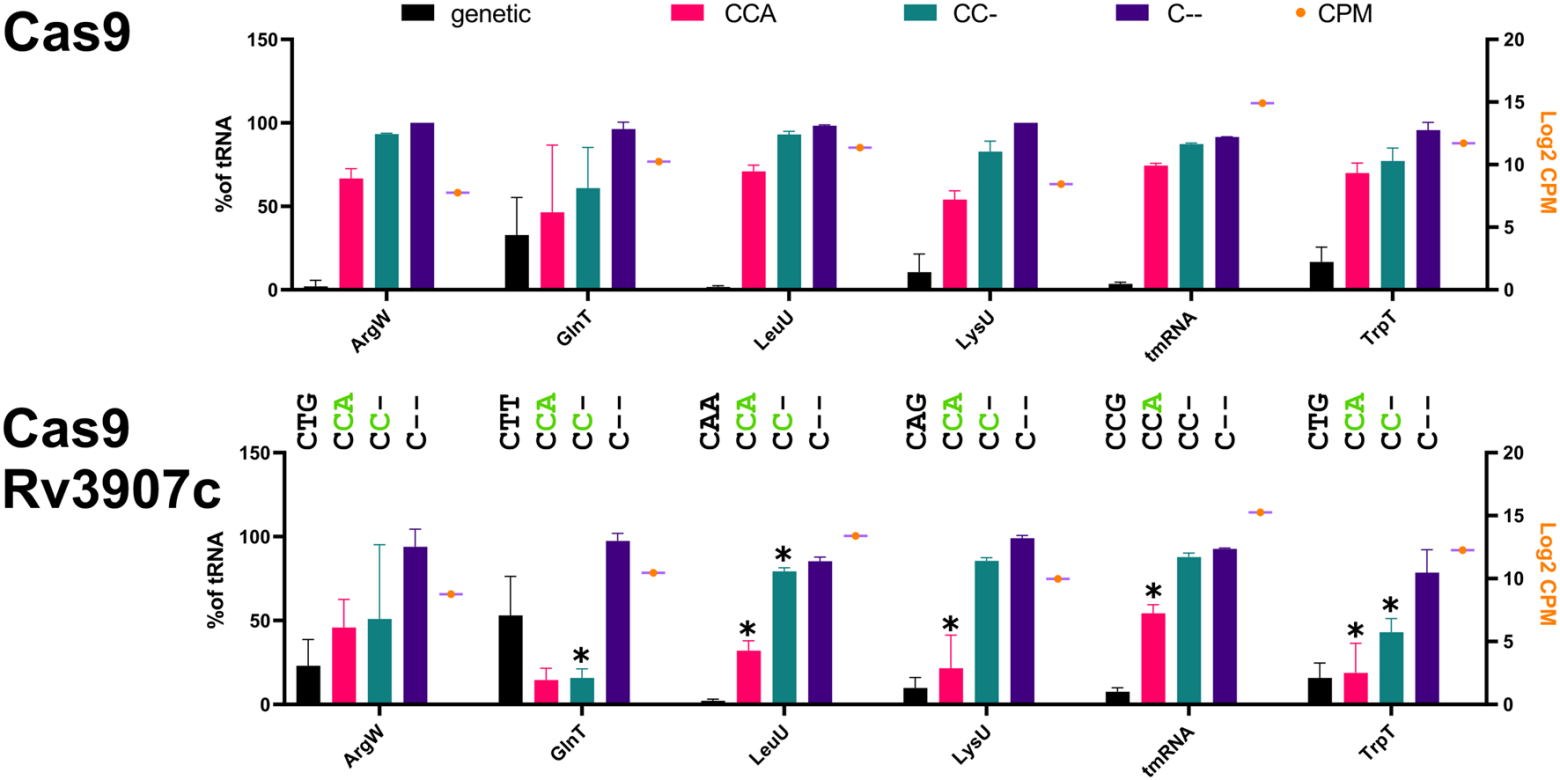
CCA adding rates upon Rv3907c downexpression**.** Quantification of tRNA species based on small RNA sequencing of Cas9 control strain and Rv3907c depletion strain. Genetic, CCA, CC-, and C- containing sequences were quantified and plotted (left axis) along with the Log2 CPM values (right axis) for each chosen tRNA and for tmRNA. CPM values are plotted as orange dots. A *p* value ≤ 0.05 was considered statistically significant.

In a separate bioinformatics analysis, we also estimated CCA maturation efficiency for all tRNAs requiting CCA synthesis and found lowered CCA addition rates for several tRNA species (best examples shown in Fig. 6).

## Discussion

The CCA-adding enzyme is essential in all organisms with at least one tRNA gene missing the genetically-encoded CCA tail; however, tRNA nucleotidyltransferase was also identified in *E. coli,* in which all tRNA genes encode the CCA tail sequence, and inactivation of this enzyme still impaired the growth of a relevant mutant strain lacking this CCA-adding enzyme^22^. Tubercle bacilli possess 45 genes encoding tRNAs, including 32 of these genes carrying no 3′-terminal CCA sequence (Table S1). Rv3907c of *Mtb* is the only recognizable member of the class II polymerase β superfamily of nucleotidyltransferases^44^, which includes poly(A) polymerases and tRNA nucleotidyltransferases/CCA-adding enzymes. The mycobacterial Rv3907c was initially annotated as probable poly(A) polymerase PcnA (polynucleotide adenylyltransferase) involved in RNA binding and RNA processing, encoded by the *pcnA* gene. Since only a fraction of tRNA genes encodes the essential, terminal CCA sequence in mycobacteria, Rv3907c is essential for the viability of bacilli, its amino acids sequence does not carry the eubacterial poly(A) polymerases-specific signature sequence but carries CCA-adding downstream motif, we propose that Rv3907c should be reannotated as CCA_*Mtb*_. The nucleobase-interacting pocket’s size and shape of EcPAP are restricted to the preferential accommodation of ATP due to the interaction of its respective body and neck domains^45^. In CCA-adding enzymes, the pocket structure accepts CMP and AMP using Asp and Arg residues which are both present in the homologous region of *Mtb* Rv3907c protein. Our in vitro experiments confirmed that CCA_*Mtb*_ presents tRNA nucleotidyltransferase activity which is inactivated by substitution of two conservative aspartic acids (DLD/57-59/ALA).

**Figure 7:**
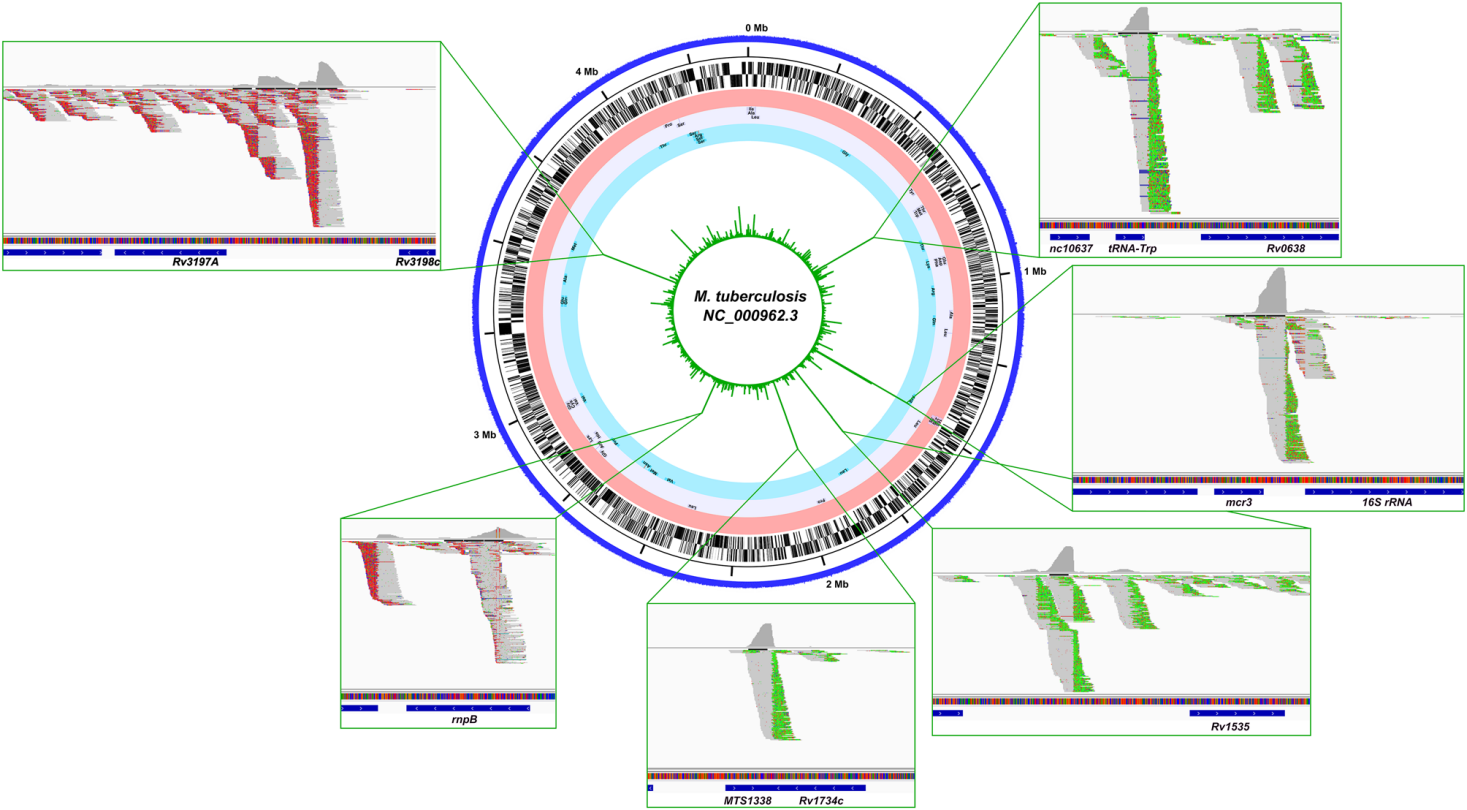
Global map of polyadenylation sites associated with CCA_*Mtb*_-depletion in *Mtb*. Total RNA sequencing files were filtered to only visualize polyA-extended sequences containing at least 8 consecutive adenines (green) or thymines (red), depending on mapping to the forward or reverse strand of the mycobacterial genome. Polyadenylated reads were abundant and strongest polyadenylation signals, depicted as screenshots from the Integrative Genomic Viewer in the Figure’s insert boxes, were recorded for noncoding RNAs: *tRNA-Trp, mcr3, MTS1338, rnpB* and for intergenic regions following genes *rv1534* and *rv3198c*. Circleator was used to produce the genomic map of polyadenylation sites^48^.

Previous studies on transcript polyadenylation in mycobacteria have remained largely inconclusive^46,47^. This was also seen by us from numerous transcriptomes analyses we have done on mycobacterial models until the present. Polyadenylated sequencing reads are nearly nonexistent in standard mycobacterial RNA Seq, and thus far, we could only detect them by specifically selecting polyadenylated reads from merged sequencing data files created from massive amounts of sequencing data. To our surprise, the major result of CCA_*Mtb*_ depletion in mycobacteria was a tremendous increase in the number of polyadenylated RNA-Seq reads, which now appeared abundant, on various RNA molecules in the cell (Fig. 7).

Polyadenylation of transcripts in bacteria is believed to be a signal for RNA decay^25,49^. We could clearly notice that among strongly polyadenylated reads are reads encoding premature, unprocessed tRNA molecules, which were strongly accumulated under CCA_*Mtb*_ depletion. Based on the heterogeneity of the polyadenylation tags, we concluded that these several-nucleotide-long additions might have in fact been produced by a different enzyme and not Rv3907c, depleted from the cell. The enzyme capable of creating such RNA tails in mycobacterial cells is PNPase, another essential enzyme implicated in both RNA maturation and degradation. We propose that CCA_*Mtb*_ deficiency affects the maturation of 3′ tRNA ends, which are then tagged for decay or further processed by PNPase as a component of RNA degradosomes^43^. The role of PNPase in abundant RNAs polyadenylation was confirmed by simultaneous silencing of both genes. The depletion of PNPase in CCA_*Mtb*_ -depleted mutant inhibited the polyadenylation of RNA transcripts. In a physiological concentration of Pi, polynucleotide phosphorylase, as a component of RNA degradosome, is involved in RNA degradation. On the other hand, it was reported that PNPase synthesizes long, highly heteropolymeric tails in vivo. In addition, RNA isolation, reverse transcription, cloning and sequencing allowed to identify the poly(A) tails in poly(A) polymerase I deficient *E. coli*^50^. It was reported that polynucleotide phosphorylase can take over the function of eubacterial polyadenylase^51,52^. It was proposed for Cyanobacteria and chloroplasts, with no identified poly(A) polymerase enzymes, that the polyadenylation is performed exclusively on PNPase in these organisms^51,52^. The in vitro poly(A) activity was also identified for PNPase of *Streptomyces coelicolor*^53^.

Interestingly, increased tRNA recycling was turned on in the CCA_*Mtb*_ depleted strain, as judged by the increased levels of transcripts encoding peptidyl tRNA hydrolase (*pth*). Pth is capable of recycling tRNA from stalled translation by cleaving the ester bond between tRNA and the peptide. As indicated in the results section of the manuscript, depletion of tmRNA molecules was also associated with CCA_*Mtb*_ downregulation in our study. Both observations indicate that the exhaustion of certain tRNA species could correlate with more frequent translation stalling. From the small RNA transcriptome data analysis, we could also see a tendency that type II RNAs were the most severely depleted of all tRNA types.

We have noticed that the mycobacterial tmRNA molecule does not encode an appropriated CCA tail, but its genetic sequence ends in CCG instead. Thus, the molecule could be a substrate for CCA-adding enzymes like typical tRNAs. Indeed, a closer inspection of sequencing traces of the tmRNA revealed that the CCG tail is often processed to CCA in the wild type strain, whereas it is either terminated at CC- or unprocessed and polyadenylated upon CCA_*Mtb*_ depletion. The lack of CCG to CCA conversion was also associated with the lack of m^1^A58-equivalent modification of the tmRNA molecules. It is likely that the tmRNA molecules present in the CCA_*Mtb*_ depletion strain remain unprocessed and nonfunctional. Since trans-translation is vital to mycobacterial cell survival, this, along with tRNA deprivation, could be the main cause of the severe loss of viability of the CCA_*Mtb*_ depleted cells.

A molecular signature of the functional CCA-adding enzyme carrying a functional HD domain is the ability to remove the products of spontaneous damage, 2′–3′ cyclic phosphates at the 3′ end of tRNAs, with its phosphatase activity^54^. Mycobacterial CCA_*Mtb*_, but not its mutated form carrying an inactivated HD domain, presented phosphatase activity as determined by pNPP suggesting its ability to fix damage at the 3′-terminal tRNA nucleotide before or after synthesis of the CCA triplet.

Overall, our study describes the role of CCA_*Mtb*_ in complex mechanisms of tRNA/tmRNA maturation and recycling and identifies a distinct regulatory program associated with CCA_*Mtb*_ depletion, which results in abundant polyadenylation of premature, unprocessed transcripts. Our work opens up a new chapter for future research on polyadenylation in *Mtb*.

## Materials and methods

### Recombinant *M. tuberculosis* CCA_*Mtb*_ expression, purification and production of anti-CCA_*Mtb*_ rabbit polyvalent serum

The *rv3907c*_*Mtb*_ gene (*rv3907c*) was amplified by PCR (all primers, vectors and strains are listed in Table S2), cloned initially in pJet1.2 (Thermo Fisher Scientific Waltham, MA USA), verified by sequencing and introduced into the pHIS parallel expression plasmid^55^ with 6-HIS N-terminal fusion. Additionally, the mutated *rv3907c*_*Mtb*_ (DLD/57-59/ALA; H/298/A) with *E. coli* optimized codons were designed, synthesized (GenScript Biotech, Leiden, Netherlands) and cloned into the expression vector pET28a. Expression of recombinant proteins was induced by 0.4 mM IPTG at 18 °C for 16 h. Bacterial cells resuspended in 10 ml of binding buffer (50 mM Tris–HCl pH 7.5, 10% sucrose, 10% glycerol, 250 mM NaCl, Sigma Aldrich, MO, USA) were disrupted using a Bioblok sonicator with a graduated LaboPlus microtip (10 cycles of 10 s with 30 s rest on ice). The resulting lysate was centrifuged 45 min at 4 °C and 16,000 RPM. The collected supernatant was purified on a HisPur Cobalt Resin agarose column (Thermo Fisher Scientific Waltham, MA USA) at 4 °C. Elution of resin bound proteins was performed using 500 mM imidazole. The His-CCA_*Mtb*_ recombinant protein was concentrated on a Novagen concentrator to a final concentration of 1 mg/ml and then used to immunize New Zealand rabbits raised under standard conventional conditions, which were approved by the Polish Ministry of Science and Higher Education Animal Facility of the Institute Microbiology, Biotechnology and Immunology, Faculty of Biology and Environmental Protection, University of Łódź. The experimental procedures were approved and conducted according to guidelines of the appropriate Polish Local Ethics Commission for Experiments on Animals No. 9 in Łódź (Agreement 9/ŁB87/2018). The immunization protocol was described previously^56^.

### tRNA nucleotidyltransferase activity

For all enzymatic assays, the His-Rv3907c and His-Rv3907c-mut recombinant proteins purified on a HisPur Cobalt Resin were further processed using 5 mL HiTrap Q FF anion exchange chromatography column (Cytiva Europe GmbH, Freiburg, Germany) calibrated 20 mM Tris–HCL pH = 8.0. The protein fractions were eluted using increasing concentrations of NaCl (100, 300, 500, 1000 mM) in 20 mM Tris–HCl pH = 8.0 and analyzed in 12% SDS-PAGE gel (Fig. S2). All enzyme assays were performed in at least three independent replicates using freshly prepared and purified enzyme in each case. 5′-fluorescently-labeled oligoribonucleotides (FAM-tRNA^Gln72^, HEX-tRNA^Val32^, HEX-tRNA^Val33AC^, HEX-tRNA^Val34ACC^) were self-annealed in 50 mM HEPES–KOH pH 7.5 and 100 mM KCl. After 2 min at 90 °C, the temperature was cooled to 37 °C, and MgCl_2_ was supplemented to a final concentration of 0.5 mM. The reactions were carried out in buffer containing 10 mM KCl, 0.1 mM EDTA, 12.5 mM MgCl_2_, 2 mM DTT, 17% glycerol, 20 mM HEPES in the presence of various concentrations of CCA_*Mtb*_, 100 nM tRNA and nucleotides (1 mM) at 37 °C for 30 min. The reactions were terminated by incubation at 95 °C for 5 min. Furthermore, proteinase K (1 µg) in 0.5 mM CaCl_2_ and 1% SDS was added to each sample and incubated at 37 °C for 30 min. Finally, loading buffer containing formamide and bromophenol blue was added to the reactions. The samples were heated at 95 °C for 5 min and then separated in a sequencing 10% polyacrylamide gel containing 8 M UREA in TBE buffer. The HEX-RNA signals were visualized using an Amersham Typhoon 8600 Imaging System.

### Metal-dependent phosphohydrolase activity

The phosphohydrolase activity protocol was prepared based on a published method^54^. Briefly, the activity was determined in 96 well microtitration plates in 200 µL reactions containing 50 mM HEPES pH 7.5, 5 mM MgCl_2_, 1 mM MnCl_2_, 0.5 mM NiCl_2_, 20 mM *p*-nithrophenyl phosphate *(p*-NPP, Sigma-Aldrich, MO, USA) 0.14–1.4 nM recombinant CCA_*Mtb*_ at 37 °C for 30 min. The reactions were terminated by the addition of 20 µl of 6 M NaOH. The p-nitrophenol produced in the reactions was detected using a BenchmarkPlus microplate spectrophotometer (Bio-Rad, Hercules, CA, USA) at 410 nM.

### Bacterial strains cultures

*E. coli* strains were cultured for 18–20 h at 37 °C in liquid or solid Luria–Bertani medium supplemented if required with 100 μg/mL ampicillin (Bioshop, Burlington, Canada). The *M. tuberculosis* strains were cultured in 7H9 or 7H10 broth (Difco, Baltimore, MD, USA) with OADC (oleic acid, albumin, dextrose, catalase; Difco, Baltimore, MD, USA) and 0.05% Tween 80 (Sigma, MO, USA) at 37 °C and were supplemented with 25 μg/mL kanamycin (Sigma, MO, USA) and/or 100 ng/mL anhydrotetracycline (aTc—Sigma, MO, USA), if required.

The essentiality of the *rv3907c* gene in *Mtb* and *Msm* strains was verified using a gene replacement protocol as previously described^38,55^. The native *rv3907c* gene was replaced with the mutated copy using a homologous recombination process exclusively in strains carrying the intact *rv3907c* gene introduced on the pMV306 integration vector into the *attB* site of the chromosomal DNA. Then, pMV306-*rv3907c* could not be replaced with pMV306 lacking intact *rv3907c* by site-specific recombination. The *rv3907c* knockdown strains were generated by the CRISPRi/dCas9 strategy^57^. The gene-specific sgRNA probes carrying approximately 20 nucleotide-long target sequences appropriately spaced from the PAM site (Table S2) were planned according to the published protocol^41^, cloned into the pLJR965 or pLJR962 plasmids, and introduced into the *M. tuberculosis* H_37_Rv or *M. smegmatis* mc^2^ 155 laboratory strains. The resulting recombinant strains carrying dCas9 and sgRNA were verified by PCR using the appropriate primers Crispr/Cas9-F and Crispr/Cas9-R (Table S2). The efficacy of translation inhibition was monitored by the growth kinetics and CCA_*Mtb*_ protein expression of tested strains in the presence of various concentrations of anhydrotetracycline and compared to the culture of *Mtb* carrying “an empty” pLJR965 plasmid. For total RNA sequencing, the strains were incubated under the abovementioned conditions for 7 days, cells were then harvested by centrifugation, and total RNA was isolated.

### Total protein isolation and Western blotting

*Mtb* cell lysates were prepared by bead beating (using 0.1 mm zirconia beads) and used for immunodetection using polyclonal antibodies raised against CCA_*Mtb*_ protein. The total protein concentration was determined by the BCA assay (Thermo Fisher Scientific Waltham, MA, USA). To compare the amount of CCA_*Mtb*_ protein in various samples, equal concentrations of the total protein were separated in sodium dodecyl sulfate–polyacrylamide gels, transferred to nitrocellulose membranes, immunodetected with anti-CCA_*Mtb*_ polyclonal antibodies using the Amersham Pharmacia ECL chemiluminescence kit and protocol, and visualized on Hyperfilm ECL (Amersham Pharmacia Biotech Ltd., UK).

### RNA isolation and sequencing

For RNA isolation, the *M. tuberculosis* wild-type strain carrying the CRISPRi/dCas9 integrative plasmid and mutants *rv3907c*^CRISPRi/dCas9^ as well as double mutant *rv3907c-gpsI*^CRISPRi/dCas9^ were grown in rich medium supplemented with anhydrotetracycline (100 ng/ml) until OD_600_ = 0.8. The cultures were then refreshed with fresh medium supplemented with anhydrotetracycline (100 ng/ml) at an OD_600_ of 0.1 and incubated in roller bottles at 37 °C. At OD_600_ 0.4–0.8, cells were spun down, and the bacterial pellet was lysed by bead beating with the MP FastPrep system using TRIzol LS reagent (Thermo Fisher Scientific Waltham, MA, USA) as described previously^58^. Three independent biological replicates were used for each strain sequenced. RNA quantity and integrity were assessed using an Agilent 2100 BioAnalyzer according to the manufacturer's instructions (Agilent RNA 6000 Nano Kit, Agilent Technologies, Inc., Santa Clara, CA, USA). RNA samples were purified (Mag-Bind TotalPure NGS, Omega Biotek, Inc. Norcross, GA, USA), and rRNA was depleted (riboPOOL, siTOOLs Biotech GmbH, Planegg/Martinsried, Germany). Three independent sequencing libraries for regular, small and poly(A)–enriched RNAs (KAPA Stranded RNA-Seq kit, KAPA Biosystems LTD, MA, USA; TrueSeq Small RNA Library Preparation Kit, Illumina Inc., San Diego, CA, USA and VAHTS mRNA-seq V3 Library Prep Kit for Illumina Inc.,Vazyme, Nanjing, People's Republic of China, respectively) were generated according to the detailed instructions provided by the producers. The quality and quantity of the resulting libraries were examined on an Agilent 2100 BioAnalyzer fitted with a DNA 1000 chip or DNA High Sensitivity chip. The NextSeq500 System (Illumina Inc., San Diego, CA, USA) with the NextSeq 500/550 Mid Output v2 sequencing kit (150 cycles, Illumina Inc., San Diego, CA, USA) was used to sequence RNA Seq libraries, ensuring 5–20 million paired-end reads per sample.

### Transcriptional analyses

The processing of RNA sequencing data was completed with a series of software and scripts as described previously^43^. Bowtie2 short read aligner^59^ or BWA-mem^60^ was used to align adapter-free reads with a minimal length of 20 bp and minimum quality of 30% to the genome of *M. tuberculosis* H_37_Rv (*NC_000962.3*, https://mycobrowser.epfl.ch/releases, Release 4 (2021–03-23), accessed on 03th of September, 2022). For data handling, converting, and indexing we applied the SAMtools software suite^61^ and Bedtools^62^. To create Fig. 7, the results of sequencing from CCA_*Mtb*_ depletion strains (6 samples) were merged and mapped, polyadenylated reads were extracted using the linux awk function. The comparison of global gene expression between the analyzed samples was performed using the default parameters of the online Degust RNA-Seq analysis platform^63^. The false discovery rate (FDR) represented the statistical analysis of differential gene expression (DGE) calculated by Degust. Differential gene expression was called when FDR < 0.05 and the log2-fold change >|2| (changing four times or more). Polyadenylation rates were estimated following BWA-mem alignment of sequencing reads, from which rRNA contaminating sequences were removed with Bazam java algorithm^63^ soft-clipped reads were pooled out with samclip (https://github.com/tseemann/samclip) and mapped reads were extracted with Bedtools. BBDuk (https://jgi.doe.gov/data-and-tools/software-tools/bbtools/bb-tools-user-guide/bbduk-guide) was used to finally quantify sequences containing “AAAAAAAA”; “AAAAAAA” and “AAAAAA”, provided as literal arguments for the search. BBDuk was also used to quantify CCA maturation in tRNA processing on small RNA libraries, where terminal 17-20nt of individual tRNAs, for all tRNAs requiring CCA maturation were quantified as literal sequences for individual genetic variants (native) and sequences ending in CCA, CC-, C- in relation to sequences devoid of CCA 3′ end.

### Supplementary Information

Supplementary Information 1.

Supplementary Information 2.

## Data Availability

The data that support this study are provided in full in the “Results” section and the Supplementary Information accompanying this paper and are available from the corresponding author upon reasonable request. The RNA/cDNA sequencing results and raw data will be made available in the Gene Expression Omnibus database (https://www.ncbi.nlm.nih.gov/geo/), before publishing. The RNA-seq related data re-used in this manuscript had been deposited to the GEO database and are accessible at https://www.ncbi.nlm.nih.gov/geo/query/acc.cgi?acc=GSE126286.
