# Supplementary material for: Depletion of tRNA CCA-adding enzyme in Mycobacterium tuberculosis leads to polyadenylation of transcripts and precursor tRNAs
Source: Sci Rep. 2023 Nov 24;13:20717. doi: 10.1038/s41598-023-47944-6 (PMC10673834; doi:10.1038/s41598-023-47944-6)
Supplement: Supplementary file 2 — Supplementary Information 2. [file 41598_2023_47944_MOESM2_ESM.pdf]

Supplementary materials

Depletion of tRNA CCA-adding enzyme in *Mycobacterium tuberculosis* leads to polyadenylation of transcripts and precursor tRNAs

Ewelina Błaszczyk[1#], Przemysław Płociński[1,2#], Ewelina Lechowicz[1], Anna Brzostek[1], Bożena Dziadek[3], Małgorzata Korycka-Machała[1], Marcin Słomka[4] and Jarosław Dziadek[1]*.

[1] Institute of Medical Biology, Polish Academy of Sciences, Lodowa 106, 93-232 Łódź, Poland,
[2] University of Łódź, Faculty of Biology and Environmental Protection, Department of Immunology and Infectious Biology, Banacha 12/16, 90-237 Łódź, Poland,
[3] University of Łódź, Faculty of Biology and Environmental Protection, Department of Molecular Microbiology, Banacha 12/16, 90-237 Łódź, Poland,
[4] University of Łódź, Faculty of Biology and Environmental Protection, Biobank Lab, Department of Oncobiology and Epigenetics, Pomorska 139, 90-235 Łódź, Poland.

# the authors contributed equally to this work

* correspondence should be addressed to jdziadek@cbm.pan.pl

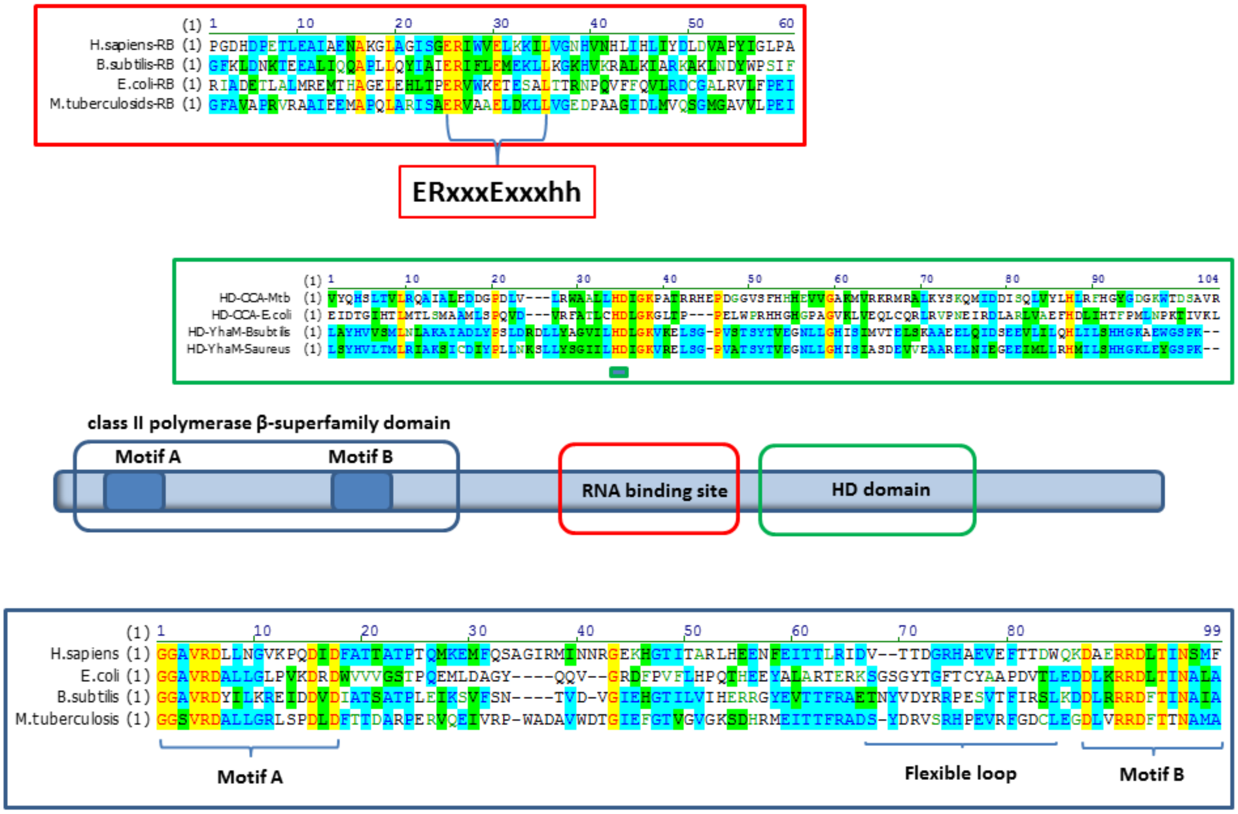

Figure S1 – The alignment analysis of CCA adding enzymes. The alignment of the regions surrounding the flexible loop in CCA adding enzymes distinguishing the tRNA nucleotidyltransferase (CCA-adding) and poly(A) polymerase representing class II polymerase β superfamily domain (blue frame). Rv3907c (*M. tuberculosis*) was compared to CCA tRNA nucleotidyltransferases of *B. subtilis* (PRK13299), *E. coli* (PRJNA612640), and Homo sapiens – mitochondrial isoform 1 (AAD34042.1). The alignment of RNA binding sites (red frame). CCA-adding enzyme consensus motif (ERxxxExxxhh) was marked (where x is any and h is hydrophobic residue). The alignment of HD domain of *M. tuberculosis* and *E. coli* CCA enzymes, YhaM exoribonucleases from *B. subtilis* (BSU_09930) and *S. aureus* (YP_500470.1). The description is based on [1] and [2]. The alignments were made using Vector NTI 11.5 software (Thermo Fisher Scientific Waltham, MA USA).

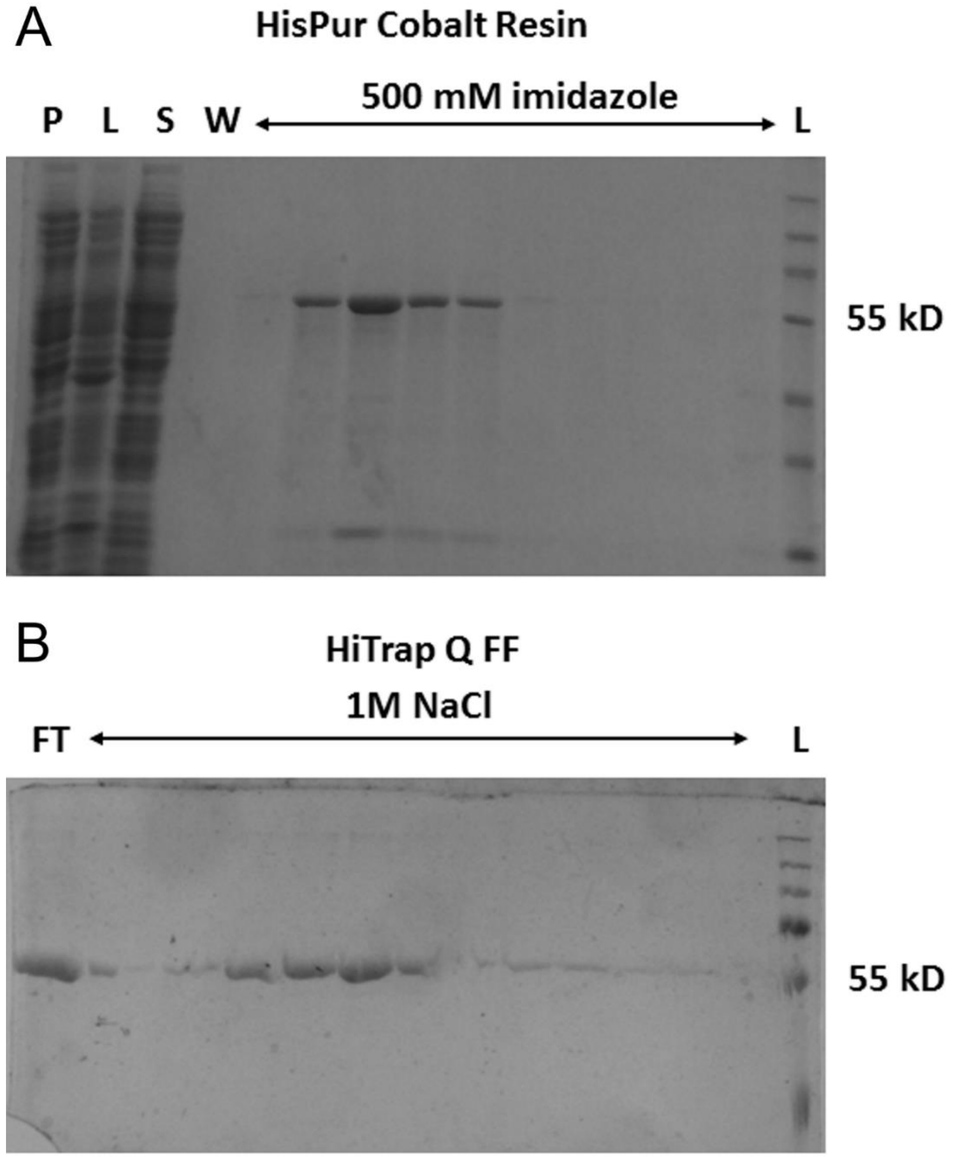

Figure S2 – The purification of Rv3907c recombinant protein. The recombined protein was purified in two steps on HisPur Cobalt Resin (**a**) and HiTrap Q FF anion exchange chromatography column (**b**). P, L, S, W, FT, L represent pellet, lysate, supernatant, wash, flow through and protein ladder, respectively. Proteins were eluted from the resin using 500 mM imidazole (**a**) and 1 M NaCl (**b**).

Original blots for figure S2

A

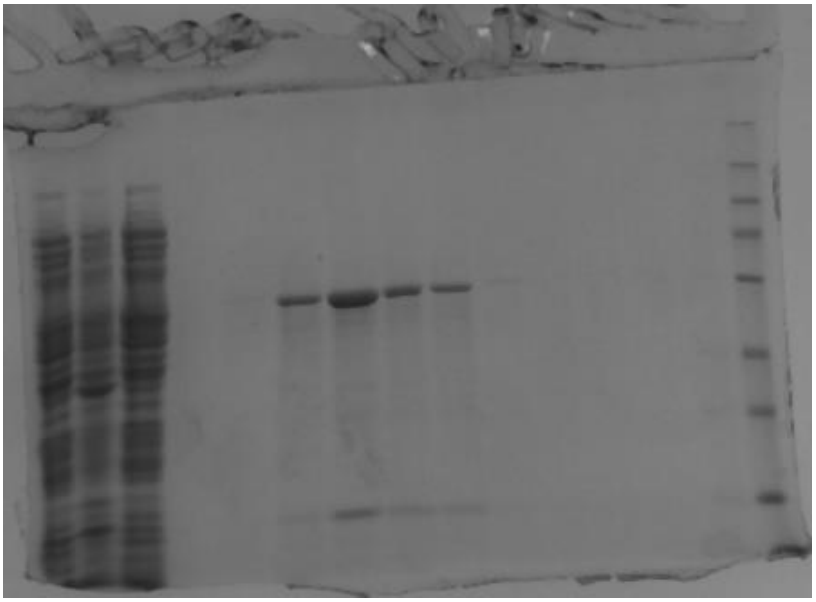

B

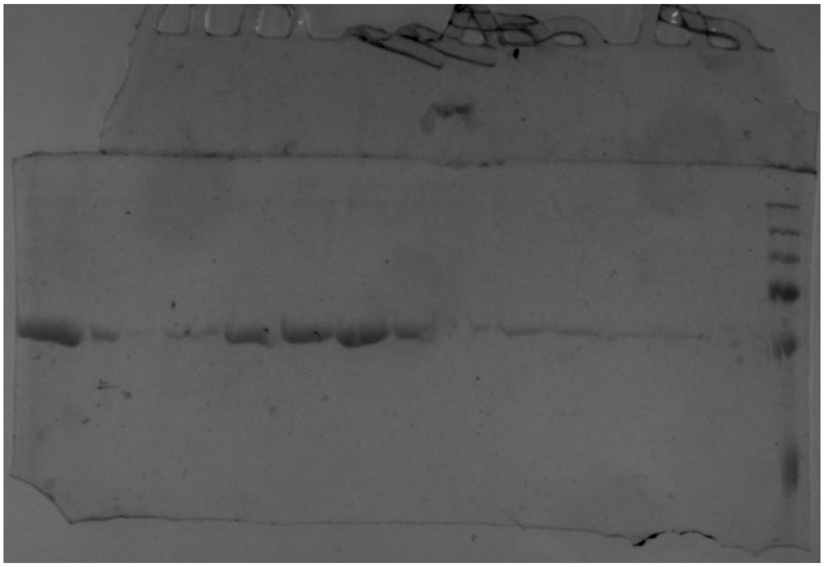

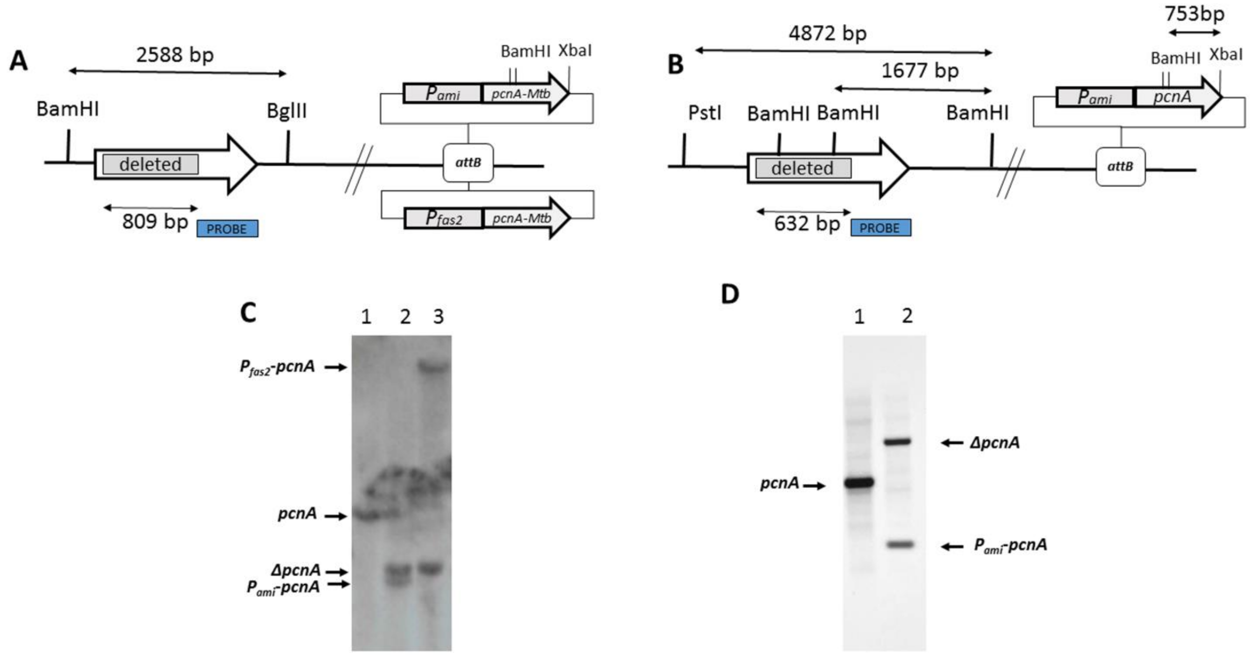

Figure S3 – Mycobacterial *pcnA* (*rv3907c*) can be inactivated exclusively in the presence of an additional, intact copy of the *pcnA* gene. (**a, b**) Genomic organization of *pcnA* genes in the *M. smegmatis ΔpcnA-pcnA* (**a**) and *M. tuberculosis* (**b**) mutants with restriction sites used for digestion of chromosomal DNA and internal deletion in Δ*pcnA*. The complementary *pcnA* controlled by P_{ami} or P_{fas2} integrated into the *attB* sites are depicted. The hybridization probes are marked by blue rectangle. (**c,d**) Southern blot hybridization of chromosomal DNA isolated from wild-type *M. smegmatis* (**c**) and *M. tuberculosis* (**d**) (Line 1) and Δ*pcnA* mutants complemented with P_{ami}-*pcnA* (Line 2) or P_{fas2}-*pcnA* (Line 3).

Original blots for figure S3

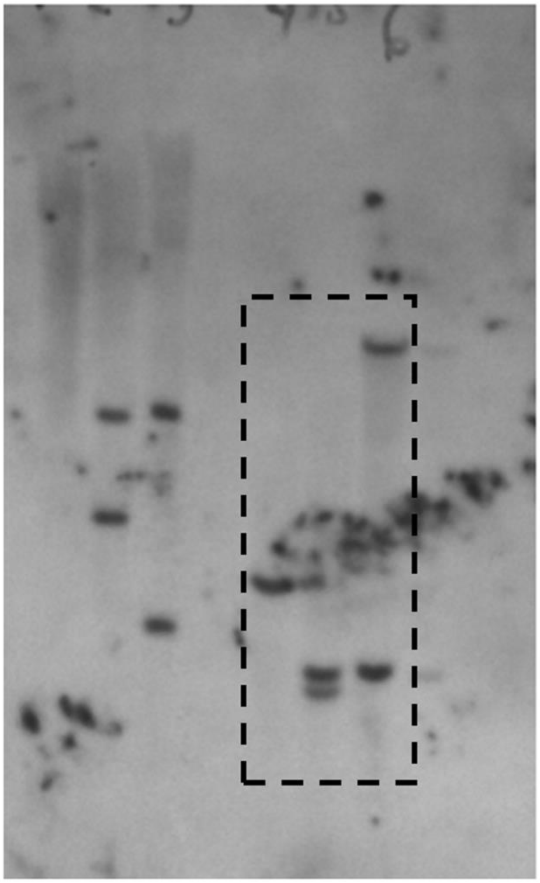

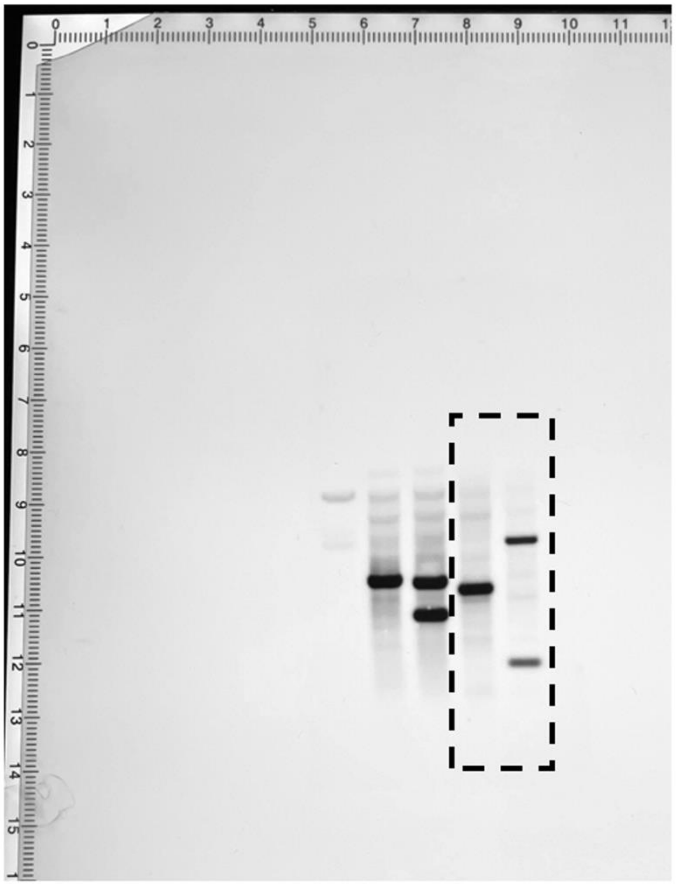

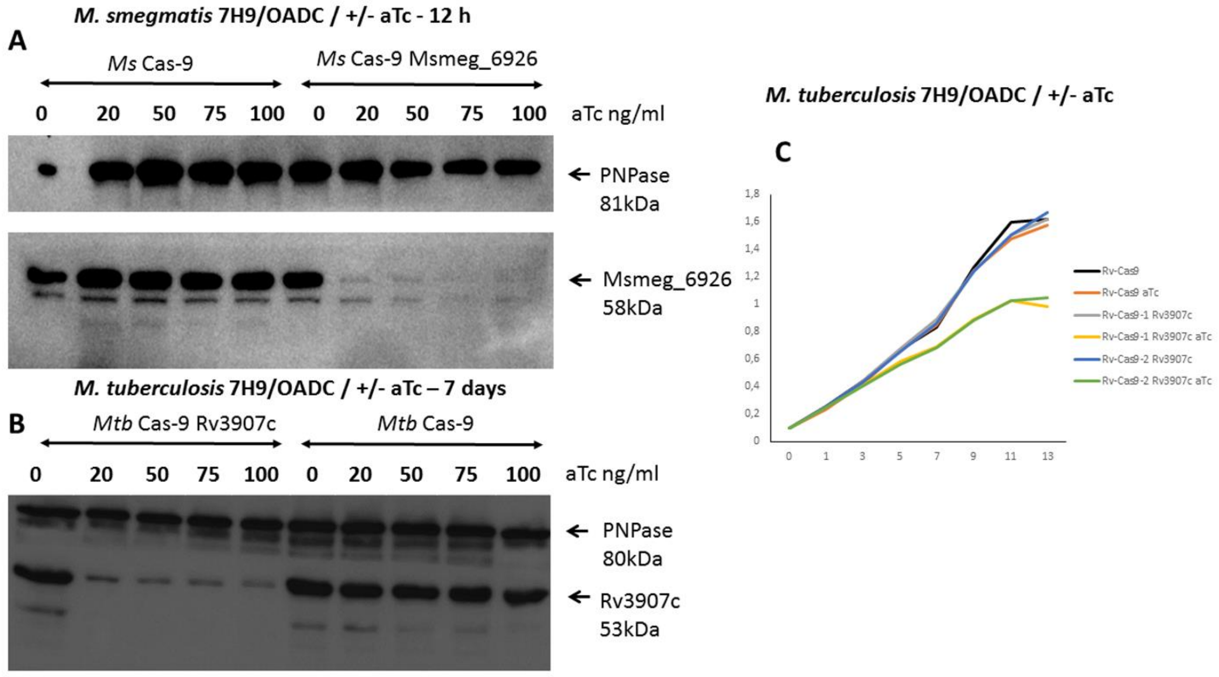

Figure S4 – Rv3907c depletion in mycobacterial *rv3907c/msmeg_6926*$^{CRISPRi/dCas9}$ mutants. Western blot analysis of total proteins isolated from (**a**) *M. smegmatis Cas-9, M. smegmatis msmeg_6926*$^{CRISPRi/dCas9}$ and (**b**) *M. tuberculosis Cas-9, M. tuberculosis rv3907c*$^{CRISPRi/dCas9}$ with polyvalent antibodies raised again Rv3907c and PNPase. The depletion of Rv3907c was induced by supplementation with aTc at the indicated concentrations. (**c**) – Rv3907c depletion-dependent growth inhibition of *M. tuberculosis* strains. Growth experiments monitored by optical density (OD_{600}) were repeated three times, with the representative result presented in the figure. Rv-Cas9 represents the control strain (*M. tuberculosis* carrying an empty CRISPRi/dCas9 vector), Cas9-Rv3907c represents *M. tuberculosis rv3907c*$^{CRISPRi/dCas9}$. The strains were cultured in rich medium 7H9/OADC and the depletion of Rv3907c/Msmeg_6926 was induced by supplementation of media with aTc (100 ng/mL).

Original blots for Figure S4

A

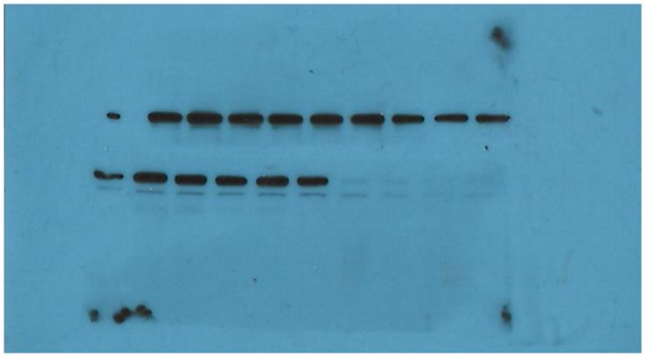

B

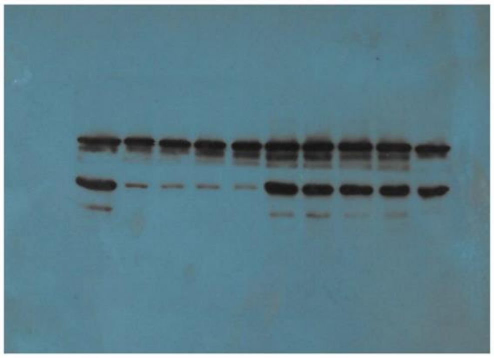

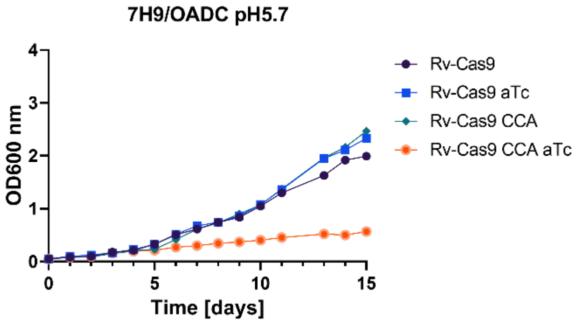

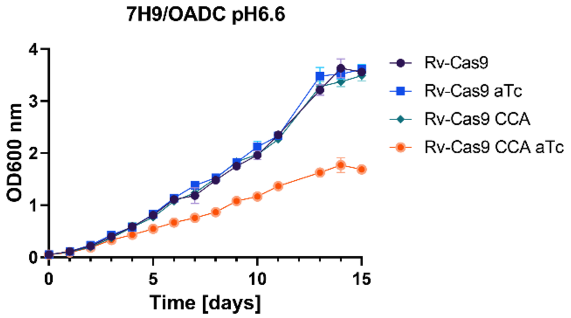

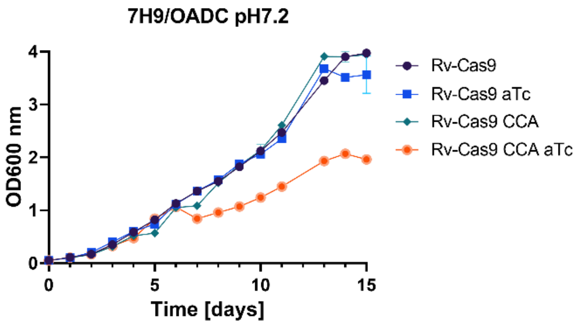

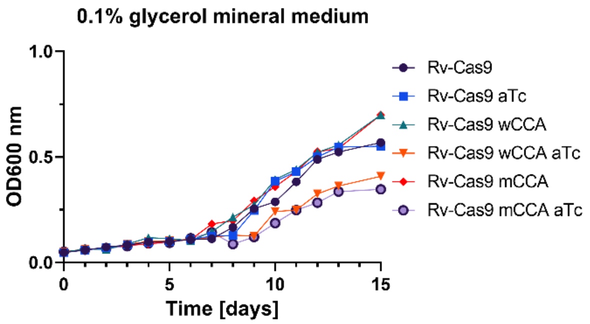

Figure S5 – Rv3907 depletion-dependent growth inhibition of *M. tuberculosis* strains in rich media (7H9/OADC) at varying pH and in minimal media. Growth experiments monitored by optical density (OD_{600}) were repeated three times, with the average (± standard deviation) presented in the figure. Rv-Cas9 represents the control strain (*M. tuberculosis* carrying an empty CRISPRi/dCas9 vector), Rv-Cas9-CCA represents *M. tuberculosis rv3907c*$^{CRISPRi/dCas9}$. The depletion of Rv3907 was induced by supplementation of media with aTc (100 ng/mL). wCCA represents weaker and mCCA medium-strength PAM resulting in 3.7-fold and 4.39-fold downregulation, respectively.

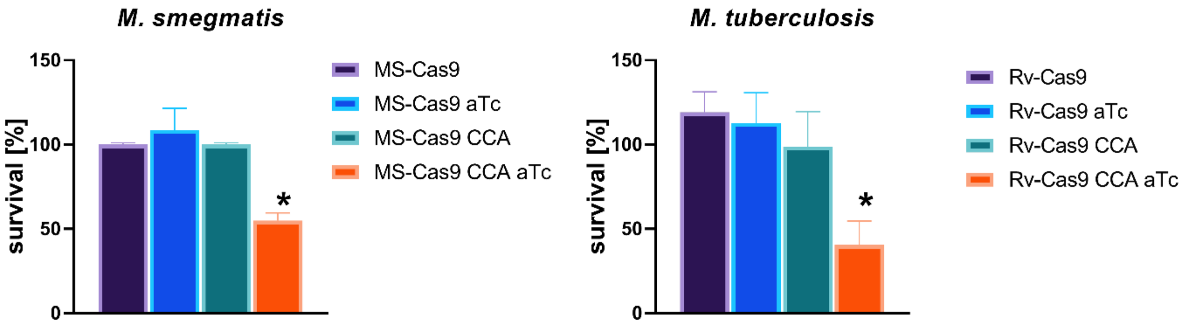

Figure S6 – Rv3907c depletion-dependent growth inhibition of *M. tuberculosis* and *M. smegmatis* strains under oxygen limitation conditions. The percent viability was calculated as the number of viable cells determined in the culture by colony forming units (CFU) under hypoxic versus normoxic conditions. MS-Cas9 and Rv-Cas9 represent control strains (*M. smegmatis* or *M. tuberculosis* carrying an empty CRISPRi/dCas9 vector, respectively), Ms-Cas9 CCA and Rv-Cas9 CCA represent corresponding *msmeg_6926/rv3907c*$^{CRISPRi/dCas9}$ strains. The depletion of Msmeg_6926/Rv3907 was induced by supplementation of media with aTc (100 ng/mL). A *t-test* was employed for comparisons of Msmeg_6926/Rv3907-depleted mutants versus the control strain to determine any significant differences between the mean values. The results were considered statistically significant (*) at $p<0.05$.

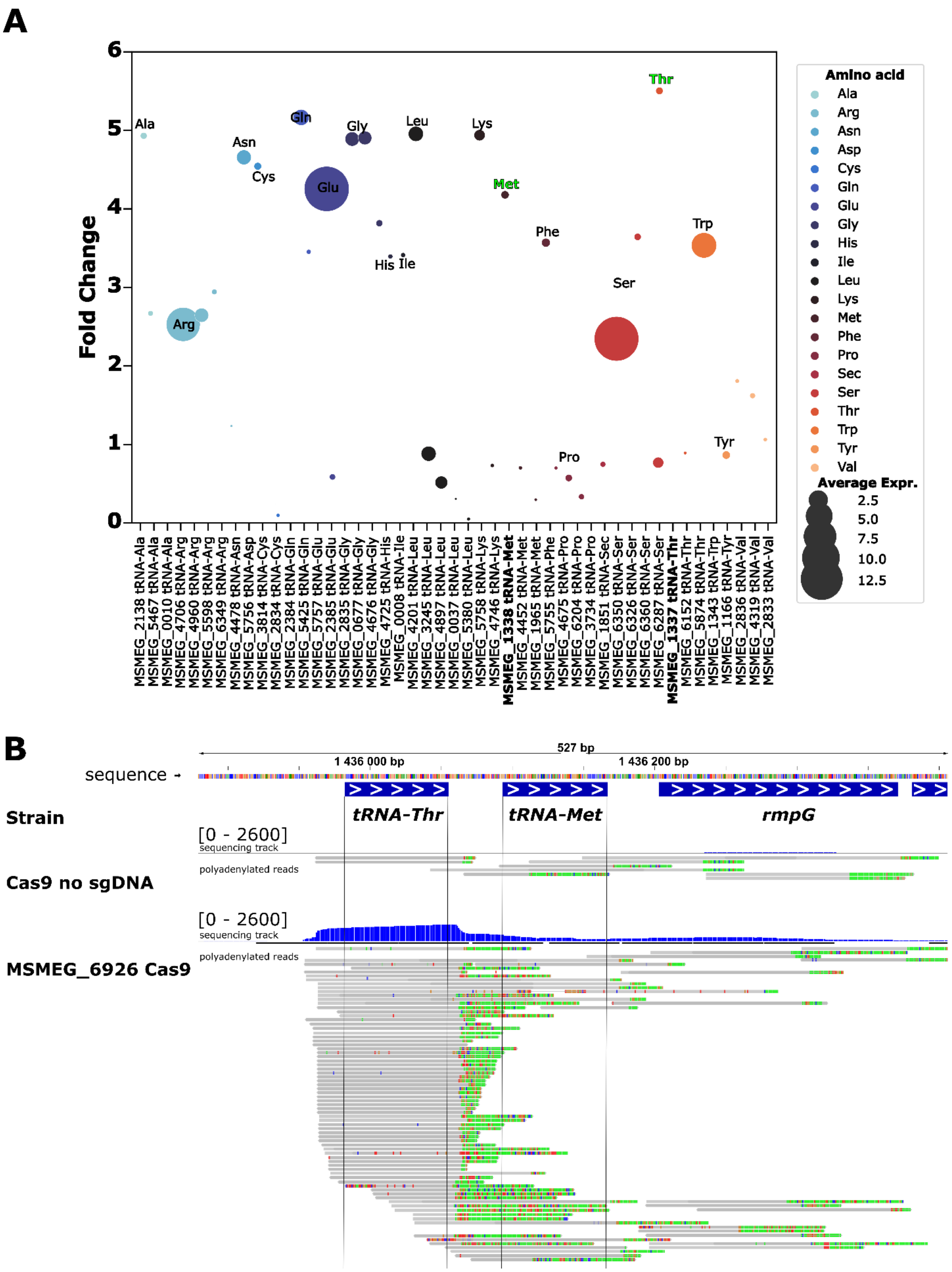

Figure S7– Log2-fold change for enrichment of tRNA molecules in cDNA libraries produced from RNA preselected on oligo-poly-dT beads from the control *M. smegmatis* strain carrying an empty Cas9 vector and Msmeg_6926 Cas9 (Msmeg_6926$^{CRISPRi/dCas9}$) strain depleted of Msmeg_6926 (**a**). Sequencing traces showing exemplary polyadenylation of transcripts encoding premature tRNA-threonine and tRNA-methionine with extra adenine residues shown in green (**b**). Sequencing traces were modified from Integrative Genomic Viewer (IGV).

Original blots for Figure 1

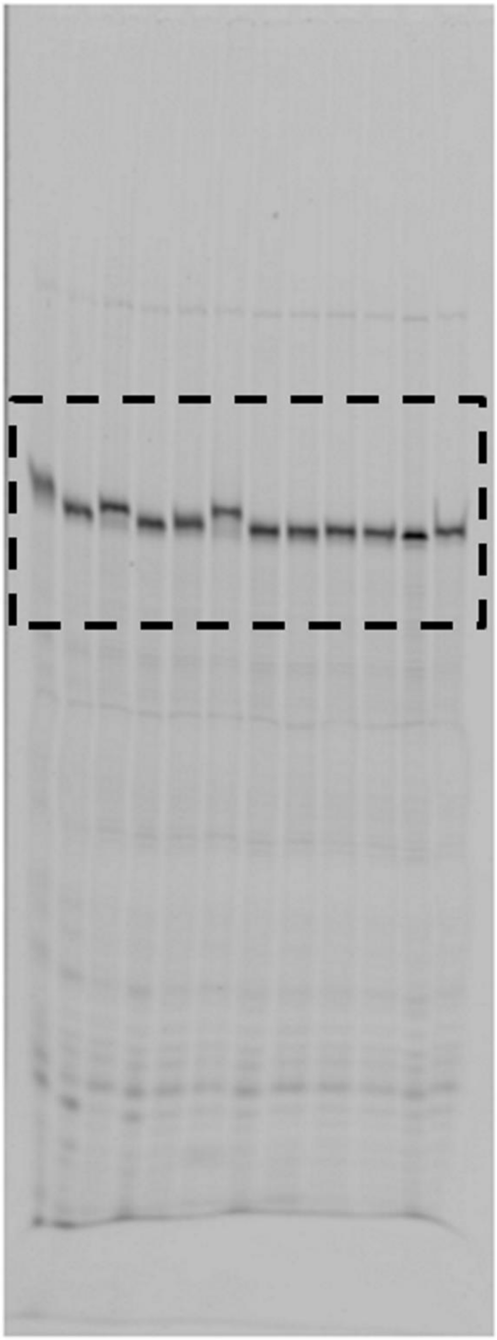

Original blot for Figure 2

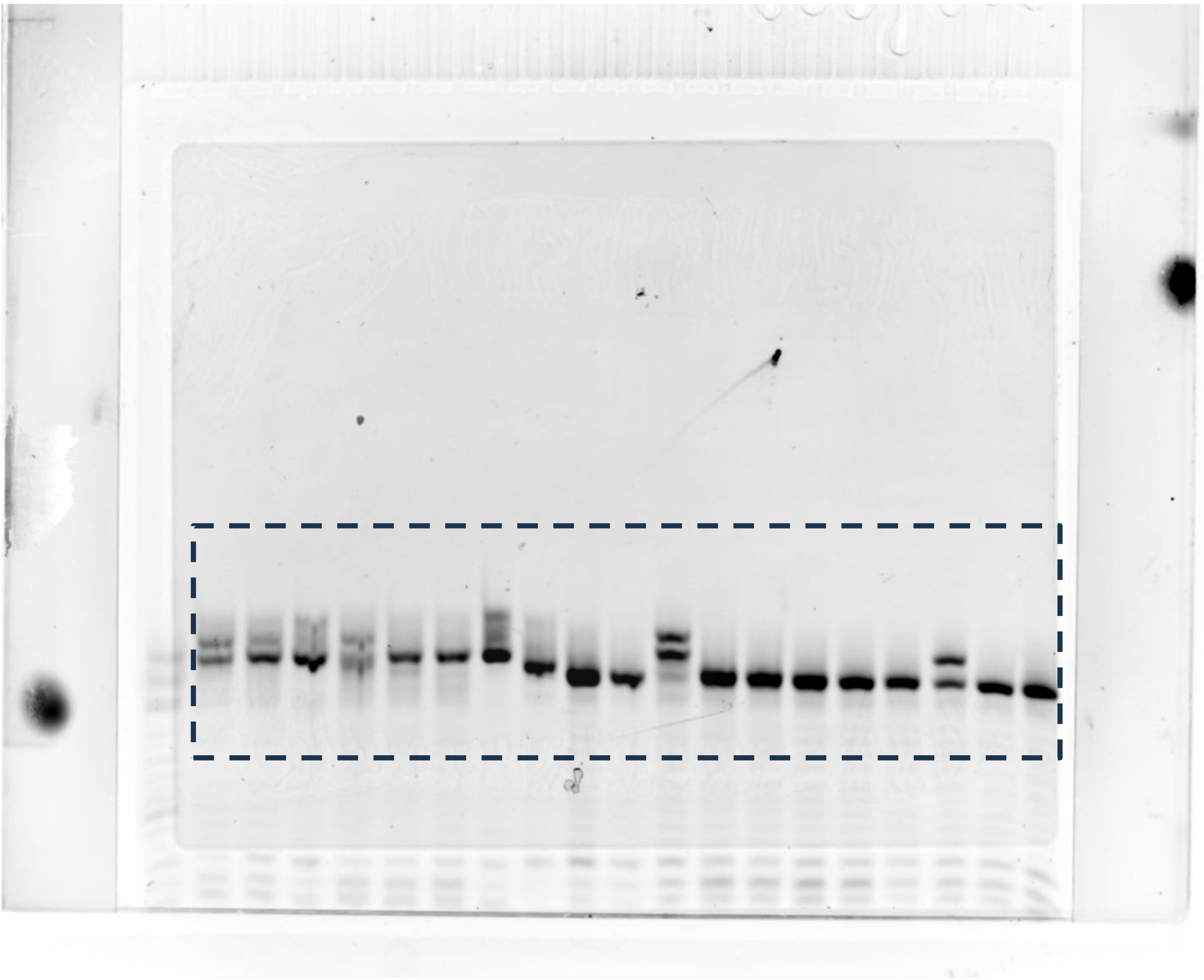

Original blot for Figure 3

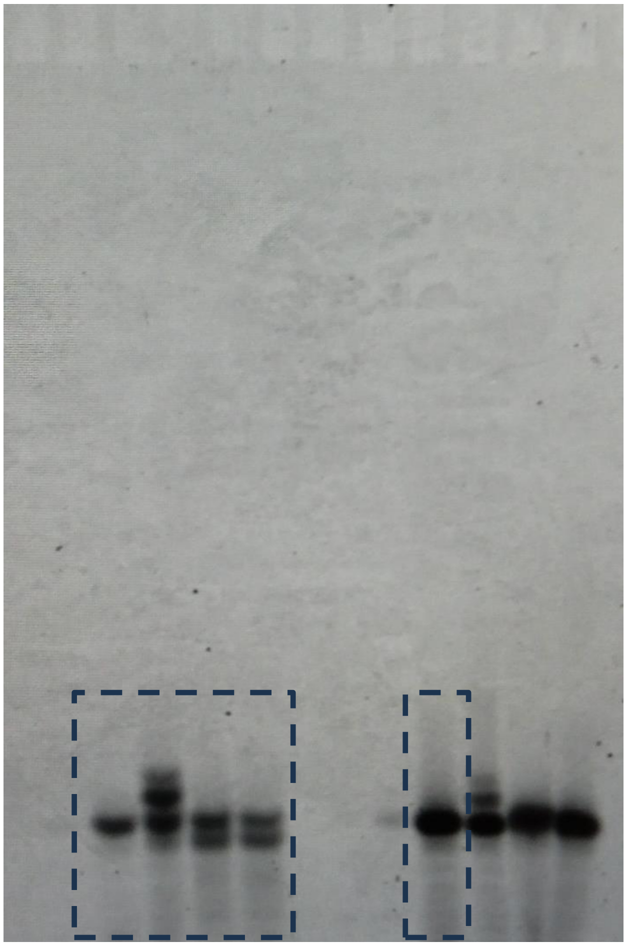

Table S1 Annotated tRNA molecules encoded by *Mycobacterium tuberculosis*

gene	name	amino acid decoding	sequence	CCA-adding	type
MTB000001	ileT	tRNA-Ile (GAT)	gggcctatagctcaggcggttagagcg cttcgctgataacgaagaggtcggagg ttcgagtcctcctaggcccacga	required	I
MTB000002	alaT	tRNA-Ala (TGC)	ggggccttagctcagttggtagagcac tgcctttgcaaggcaggggtcaggggt tcgagtcccctaggctccacaa	required	I
MTB000003	leuT	tRNA-Leu (CAG)	gggcgagtggcggaatggcagacgcg ctggcttcaggtgccagtgtccttcggg acgtgggggttcaagtccccccttcgccc accg	required	II
MTB000004	glyU	tRNA-Gly (CCC)	gccgatgtagttcaatggcagaacatc agcttcccaagctgaatacgcgggttc gattcccgtcatcggctccg	required	I
MTB000005	tyrT	tRNA-Tyr (GTA)	ggcaggttgcccgagcggccaatggg agcggactgtaaatccgtcgcgaaagc tacgcaggttcgaatcctgcacctgcc acca	not required	II
MTB000006	thrT	tRNA-Thr (GGT)	gccccttagctcagtcggcagagcgt ttccatggtaaggaaaaggtcaacggt tcgattccgttagggggctcgg	required	I
MTB000007	metT	tRNA-Met (CAT)	ggcgatgtagctcagtcggttagagcg aacgactcataatcgttaggtcgccgg ttcgagtccggccatcgctacaa	required	I
MTB000008	trpT	tRNA-Trp (CCA)	aggggcgtagctcaactggcagagca gcggtctccaaaaccgcaggttgcagg ttcaagtcctgtcgcccctgctg	required	I
MTB000009	thrV	tRNA-Thr (TGT)	gcctccttagctcagtggtagagcactc gccttgtaagcgagcggtcgtcagttc aatcctgacagggggctcaa	required	I
MTB000010	lysT	tRNA-Lys (TTT)	gcccctatagctcagttggtagagctac ggacttttaatccgcaggtcccaggttc gagtcctggtgggggcacaa	required	I
MTB000011	gluT	tRNA-Glu (TTC)	gcccccttcgtctagacggcctaggac gccgccctttcaaggcggtaacgcggg ttcgaatcccgtagggggtacct	required	I
MTB000012	aspT	tRNA-Asp (GTC)	ggccctgtggcgcagttggttagcgcg ccgccctgtcacggcggaggtcgcggg ttcgagtcccgtcagggtcgcca	not required	I
MTB000013	pheU	tRNA-Phe (GAA)	ggccaggtagctcagtcggtatgagcg tccgcctgaaaagcggaaggtcggcg gttcgatcccgcccctggccacca	not required	I

MTB000014	argT	tRNA-Arg (CCT)	gccctcgtagctcaggggatagagcacggctctcctaaagccggtgtcgcaggttcgaatcctgccgggggcactc	required	I
MTB000015	alaV	tRNA-Ala (CGC)	ggggctatggcgcagttggtagcgcgactcgttcgcatcgagtaggtcaggggttcgaatccccttagctccacca	not required	I
MTB000016	glnT	tRNA-Gln (TTG)	tccgtcgtggtgtaatcggcagcacctctgattttggttcagatagttcaggttcgagtcctggcgacggagctt	required	I
MTB000017	leuX	tRNA-Leu (TAA)	gcccccatagcccaattggcagaggcagcggacttaaaatccgtcaagtgtcggttcgagtccgactgggggcacgg	required	I
MTB000018	argV	tRNA-Arg (CCG)	gcccccgtagctcaggggatagagcgtctgcctccggagcagaaggccgcaggttcgaatcctgccgggggcacaa	required	I
MTB000022	leuW	tRNA-Leu (TAG)	gcgggcgtgatgaaattggcaaacatgccggttttaggtgccggtgctcgaaagagtttgagggttcgagtccctccgcccgcactc	required	II
MTB000023	leuV	tRNA-Leu (CAA)	gccctcgtatcccaactggcagaggaaacggactcaaaacccgtccagtgtgggttcgaatcccaccgagggcacca	not required	I
MTB000024	proT	tRNA-Pro (GGG)	cgggctgtggcgcagtttggtagcgcacttgactgggggtcaagtggtcgcaggttcaaatcctgtcagcccgactt	required	I
MTB000025	leuU	tRNA-Leu (GAG)	gtccgagtggcggaatggcagacgcgctagcttgaggtgctagtgccctactaatgggcgtgggggttcaagtccccccctcggacacaa	required	II
MTB000027	valV	tRNA-Val (TAC)	gggcgcgtagctcagcggtagagctctggttttacacaccagcggtcggcggttcgatcccgtccgcgcccaccg	required	I
MTB000028	metV	tRNA-Met (CAT)	ggggcggtagctcagttggttagagccgcggactcataatccgttggtcgcgggttcgagccccgcccgccccacat	required	I
MTB000029	asnT	tRNA-Asn (GTT)	tcccctgtagctcaattggcagagcgttcggctgttaaccgaagggttggaggttcgagtcctcccgggggagcag	required	I
MTB000030	proU	tRNA-Pro (TGG)	cggggtgtagcgcagcttggtagcgcatccgctttgggagcggaaggccgcaggttcaaatcctgtcaccccgacca	not required	I
MTB000031	glyV	tRNA-Gly (TCC)	gcgggcgtagctcaatggtagagccctagtcttccaaactagcgacgcgggttcgattcccgtcgcccgctcgg	required	I
MTB000032	argW	tRNA-Arg (TCT)	gcctccgtagctcaggtggatagagcaagggccttctaatccctaggtcgcacgttcgagtcgtgccgggggcactg	required	I

MTB000033	hisT	tRNA-His (GTG)	gtgagtgtagttcagttggtagagcac caggttgtgatcctgggtgtcgcgggtt cgagtcccgtcactcacccca	not required	I
MTB000034	lysU	tRNA-Lys (CTT)	gcgccgttagctcagttggtagagcag ctgactcttaatcagcgggtccggggtt cgaaaccctgacggcgcacag	required	I
MTB000035	valT	tRNA-Val (CAC)	ggtcccgtggctcagtgggagagcgtc cgcttcacacgcggaaggtcgctggtt cgatcccagccgggaccacta	required	I
MTB000036	glyT	tRNA-Gly (GCC)	gcggatgtagcgcagttggtagcgcat caccttgccaaggtgagggtcgcgggt tcgaatcccgtcatccgctcga	required	I
MTB000037	cysU	tRNA-Cys (GCA)	ggtggagtggccgagtggtgaggcaa cggcctgcaaagccgtgcacacgggtt cgattcccgtctccacctcca	not required	I
MTB000038	valU	tRNA-Val (GAC)	gcgcgattagctcagcgggagagcgct tccctgacacggaagaggtcactggtt caatcccagtatcgcgcacca	not required	I
MTB000039	gluU	tRNA-Glu (CTC)	ggccccgtcgtctagcggcctaggacg ccgccctctcacggcggtagcgtgggtt cgaatcccatcggggctacaa	required	I
MTB000040	glnU	tRNA-Gln (CTG)	tggggtatggtgtaattggcaacacag ctgattctggttcagccattctaggttcg agtcctggtaccccagcac	required	I
MTB000041	alaU	tRNA-Ala (GGC)	ggggctatggcgcagctggtagcgcac cacactggcagtgtgggggtcaggggt tcgagtccccttagctccactc	required	I
MTB000043	metU	tRNA-fMet (CAT)	cgcggggtggagcagctcggtagctcg ctgggctcataacccagaggtcgcagg ttcgaatcctgtccccgctacca	not required	I
MTB000044	thrU	tRNA-Thr (CGT)	gccgccttagctcagtcggtagagcga ttcactcgtaatgaataggtcaggagtt cgattctcctaggcggctcca	not required	I
MTB000045	proY	tRNA-Pro (CGG)	cggggtgtggcgcagcttggtagcgcg cttcgttcgggacgaagaggccgtggg ttcaaatcccgccaccccgaccg	required	I
MTB000046	serV	tRNA-Ser (GGA)	ggaggattcgcctagtggcctatggcg ctcgcctggaacgcgggttgggttaac agccctcgcgggttcaaatcccgcatc ctccgcca	not required	II
MTB000047	serX	tRNA-Ser (CGA)	ggtggcgtgtccgagcggcctaagga gcacgcctcgaaagcgtgtgacggcta acaccgtccgagggttcaaatccctcc gccaccgcca	not required	II
MTB000048	argU	tRNA-Arg (ACG)	gcgcccgtagctcaacggatagagcat ctgactacggatcagaaggttgggagt tcgaatctcttcgggcgcgctc	required	I

MTB000049	serT	tRNA-Ser (GCT)	ggaggcgtgccagagcggccgaatgg ggctcactgctaatgagttgtccccctt caaggggaccggaggttcaaatcctct cgcctccgcag	required	II
MTB000050	serU	tRNA-Ser (TGA)	ggtggcgtggcagagcggcctaatgc actcgccttgaaagcgagagacggcta acaccgtccgggggttcaaatccctcc gccaccgctg	required	II
MTB000042 tmRNA	ssr	10Sa RNA	ggggctgaacggtttcgacttcgcgca tcgaatcaagggaagcgtgccggtgc aggcaagagaccaccgtaagcgtcgtt gcgaccaaataagcgccgattcacatc agcgcgactacgctctcgctgcctaag cgacggctagtctgtcagaccgggaac gccctcggcccggaccctggcatcagc tagagggatccaccgatgagtccggtc gcgggactcctcgggacaaccacagc gactgggatcgtcatctcggctagttcg cgtgaccgggagatccgagcagaggc atagcgaactgcgcacggagaagcctt gagggaatgccgtaggacccgggttc gattcccggcagctccaccg	required	tmRNA

Table S2 List of plasmids, strains and oligonucleotides used, annotated tRNAs.

**oligonucleotides used in this study**		
Name	Sequence (5'>3')	Application
	**Primers used for cloning**	Amplification region
RvpcnAGR1Kpn-nat	cggTACCGTGAGCGCCATCCCGAG	*Mtb-pcnA* upstream region (1327bp) including 5' of *pcnA* gene (119 bp)
RvpcnAGR2HindIII	gaagctTACAACTCGTGTCCCGCGGCG	
RvpcnAGR3HindIII	gaagcttAAATCGGTGGGATGCGGATGG	*Mtb-pcnA* downstream region (1558 bp) including 3' of *pcnA* gene (692 bp)
RvpcnAGR4PstI	gctgcaGTTGTGTTGATAACCCGCACCGG	
MsmegpcnAGR1PstI	cctgcagCGCGCTGCATCTCGGTGG	*Msmeg-pcnA* upstream region (1337 bp) including 5' of *pcnA* gene (134 bp)
MsmegpcnAGR2HindIII	cggatccGAGCCTAATGCGCGCCGC	
MsmegpcnAGR3HindIII	gaagcttAGGACGTGTACTGGCACTCGCTGAC	*Msmeg-pcnA* downstream region (1247 bp) including 3' of *pcnA* gene (634 bp)
MsmegpcnAGR4KpnI	cggtaccGGCTCGTCAGTTCGTTCGAGTGTTG	
MtbpcnAsBglII	gagatctGTGCCGGAAGCCGTCCAGGAAG	*Mtb-pcnA* gene to clone into pJam2 vector under control of P_{ami} promoter
MtbpcnArXbaI	ctctagaCACAACCGTTCTGACGCGACTCCC	
Rvpap1HISBglII-s	cagatctgGTGCCGGAAGCCGTCCAGGAAG	*Mtb-pcnA* gene to clone into pHIS expression vector
Rvpap1HISHindIII-r	caagcTTAGCGGTTCCCCCGTGATTTCCA	
RvPAPI-mutSma1bsen	acccggGGCAAGCCCGCCACCCGC	Inner primers of *Mtb-pcnA* used to mutate HD domain
RvPAPI-mutSma1rev	acccgggcggcCAGCAGCGCCGCCCAGCG	
Ms-pcnA-fas2-Xba-s	ctctagatATGACCACGGCGCCGGCCTC	*Msmeg-pcnA* gene to clone into pMV306-Pfas2 under P_{fas2} promoter
Ms-pcnA-fas2-EcoRI	cgaattcTCAGCCTCTCGCGTTCCACCATTTC	
**oligonucleotides used for gene silencing**		
MsCasPAPI158-for	gggaACAGCGCGTCGGACCAGCCGC	Oligonucleotide cloned into pLJR962 vector (*Msmeg-pcnA* silencing), PAM 158x NNAGAAA
MsCasPAPI158-rev	aaacGCGGCTGGTCCGACGCGCTGT	
MtbCasPAP84-for	gggaACCCGAGTTCCCGCAATAAG	Oligonucleotide cloned into pLJR965 vector (*Mtb-pcnA* silencing), PAM 84.6 NNAGCAT
MtbCasPAP84-rev	aaacCTTATTGCGGGAACTCGGGT	
MtbCasPAP42-for	gggaGTTCAAGGCAACCGCAGCGGCGG	Oligonucleotide cloned into pLJR965 vector (*Mtb-pcnA* silencing), PAM 82.2 NNAGCAG
MtbCasPAP42-rev	aaacCCGCCGCTGCGGTTGCCTTGAAC	
Cas9_PNPtb_sPAM_F	gggaGCTCCTCGACGTCGACCGTGAGGGG	

Cas9_PNPtb_sPAM_R	aaacCCCCTCACGGTCGACGTCGAGGAGC	Oligonucleotide cloned into pLJR965 vector carrying pcnA$^{CRISPRi/dCas9}$ (*Mtb-pcnA/gpsI* silencing), PAM 216.7 NNAGAAG
Crispr/Cas9-F	GATCGAGATGGCCCGCGAGA	to confirm the integration of CRISPR/Cas9 in mycobacteria
Crispr/Cas9-R	CACGGCGTGGTGGTGGTAGGT	
**oligonucleotides used in PAPI activity assays**		
tRNA72-Gly	[56FAM]UGGGGUAUGGUGUAAUUGGCAACACAGCUGAUUCUGGUUCAGCCAUUCUAGGUUCGAGUCCUGGUACCCCAG	*in vitro* tRNA nucleotidylotransferase assay
tRNA_CC	[HEX]GGGUGAUGGCGGUUCGAUCCCGUCAUCACCCACC	*in vitro* tRNA nucleotidylotransferase assay
tRNA_CC	[HEX]GGGUGAUGGCGGUUCGAUCCCGUCAUCACCCAC	*in vitro* tRNA nucleotidylotransferase assay
tRNA_0	[HEX]GGGUGAUGGCGGUUCGAUCCCGUCAUCACCCA	*in vitro* tRNA nucleotidylotransferase assay
**Strains used in this study**		
**Name**	**Description**	**Reference**
Top10F'	*Escherichia coli* strain	Invitrogen
Mc^{2}155	*M. smegmatis* wild type	Laboratory stock
BL21 (DE3) pLysS	*E. coli* strain	Novagen
H37Rv	virulent strain of *M. tuberculosis*	Laboratory stock
Δ*pcnA*-P_{ami}*pcnA*	*M. smegmatis* Δ*pcnA* mutant, carrying *pcnA* with internal deletion, complemented with *pcnA* gene under P_{ami} promotor in *attB* site	This study
Δ*pcnA*-P_{ami}*pcnA*	*M. tuberculosis* Δ*pcnA* mutant, carrying *pcnA* with internal deletion, complemented with *pcnA* gene under P_{ami} promotor in *attB* site	This study
Δ*pcnA*-P_{fas2}*pcnA*	*M. tuberculosis* Δ*pcnA* mutant, carrying *pcnA* with internal deletion, complemented with *pcnA* gene under P_{fas2} promoter in *attB* site	This study
Msmeg pcnA$^{CRISPRi/dCas9}$	*M. smegmatis* mutant with pLJR962 plasmid carrying *pcnA*-silencing sequence	This study
Msmeg$^{CRISPRi/dCas9}$	*M. smegmatis* carrying pLJR962 plasmid	This study
Mtb pcnA$^{CRISPRi/dCas9}$	*M. tuberculosis* mutant with pLJR965 plasmid carrying *pcnA*-silencing sequence	This study
Mtb$^{CRISPRi/dCas9}$	*M. tuberculosis* carrying pLJR965 plasmid	This study
**Plasmids used in this study**		
	**Vector description**	
pJET 1.2/blunt	Blunt cloning vector, Amp^R, for subcloning of gene fragments	Thermo Scientific

p2NIL	Suicidal gene delivery vector, nonreplicating in mycobacteria, Kan^R	Parish& Stoker, 2000
pGoal17	Source of PacI cassette, Amp^R	Parish& Stoker, 2000
pMV306K	Mycobacterial integrating vector, Kan^R	Med-Immune Inc
pMV306H	Mycobacterial integrating vector, Hyg^R	Med-Immune Inc
pHIS	*E. coli* expression vector enabling the purification of HIS-tagged protein, Amp^R	Sheffield et al., 1999
pET28a(+)	*E. coli* expression vector enabling the purification of His-tagged protein, Km^R	Amersham Biosciencies
p2NIL-GR*pcnA*$_{Mtb}$	p2NIL carrying Δ*pcnA*$_{Mtb}$ with flanking regions and PacI cassette from pGOAL17	This study
p2NIL-GR*pcnA*$_{Msmeg}$	p2NIL carrying Δ*pcnA*$_{Msmeg}$ with flanking regions and PacI cassette from pGOAL17	This study
pHIS-*pcnA*$_{Mtb}$	pHIS carrying *pcnA*$_{Mtb}$	This study
pET28-*pcnA*$_{Mtb}$-mutDLD/ALA (DLD/57-59/ALA)	pET28a carrying *pcnA*$_{Mtb}$ -(DLD/57-59/ALA) – *E.coli* optimized codons	This study
pET28-*pcnA*$_{Mtb}$- mutH/A (H/298/A)	pET28a carrying *pcnA*$_{Mtb}$ -(H/298/A) – *E.coli* optimized codons	This study
pMV306H- P_{ami}*pcnA*	Mycobacterial integrating vector carrying *M. tuberculosis pcnA* under P_{ami} promoter	This study
pMV306H- P_{fas2}*pcnA*	Mycobacterial integrating vector carrying *M. tuberculosis pcnA* under P_{fas2} promoter	This study

1 Hoffmeier, A. *et al.* Unusual evolution of a catalytic core element in CCA-adding enzymes. *Nucleic Acids Res* **38**, 4436-4447 (2010). https://doi.org/10.1093/nar/gkq176

2 Martin, G. & Keller, W. Sequence motifs that distinguish ATP(CTP): tRNA nucleotidyl transferases from eubacterial poly(A) polymerases. *Rna* **10**, 899-906 (2004). https://doi.org/Doi 10.1261/Rna.5242304
